# Impact of the DREAMS Partnership on social support and general self-efficacy among adolescent girls and young women: causal analysis of population-based cohorts in Kenya and South Africa

**DOI:** 10.1136/bmjgh-2021-006965

**Published:** 2022-03-01

**Authors:** Annabelle Gourlay, Sian Floyd, Faith Magut, Sarah Mulwa, Nondumiso Mthiyane, Elvis Wambiya, Moses Otieno, Vivienne Kamire, Jane Osindo, Natsayi Chimbindi, Abdhalah Ziraba, Daniel Kwaro, Maryam Shahmanesh, Isolde Birdthistle

**Affiliations:** 1 Faculty of Epidemiology of Population Health, London School of Hygiene and Tropical Medicine, London, UK; 2 Center for Global Health Research, Kenya Medical Research Institute, Kisumu, Kenya; 3 Health and Systems for Health, African Population and Health Research Center, Nairobi, Kenya; 4 Institute for Global Health, University College London, London, UK; 5 Africa Health Research Institute, KwaZulu-Natal, South Africa; 6 University of KwaZulu-Natal, KwaZulu-Natal, South Africa

**Keywords:** cohort study, epidemiology, health systems evaluation, prevention strategies, HIV

## Abstract

**Introduction:**

The Determined, Resilient, Empowered, AIDS-free, Mentored and Safe (DREAMS) Partnership aimed to influence psychosocial processes that promote empowerment among adolescent girls and young women (AGYW), and reduce HIV incidence. We estimated the impact of DREAMS on aspects of AGYW’s collective and individual agency (specifically, social support and self-efficacy), in three settings where DREAMS was implemented from 2016 until at least end 2018.

**Methods:**

Research cohorts of ~1500 AGYW aged 13–22 were randomly selected from demographic platforms in Kenya (Nairobi; Gem) and South Africa (uMkhanyakude) and followed up from 2017 to 2019. Social support was based on questions about female networks and access to safe places to meet with peers; general self-efficacy was measured using a scale previously validated in other settings. We conducted multivariable logistic regression, and estimated the causal effect of invitation to DREAMS on each outcome in 2018 and 2019 by comparing counter-factual scenarios in which all, vs no, AGYW were DREAMS invitees.

**Results:**

In Nairobi, Gem and uMkhanyakude, respectively, 74%, 57% and 53% were invited to DREAMS by 2018. Social support was higher among DREAMS invitees versus non-invitees (eg, adjusted OR 2.0 (95% CI 1.6 to 2.6), Gem, 2018). In 2018, DREAMS increased social support in all settings and age groups, for example, from 28% if none were DREAMS invitees to 43% if all were invitees (+15% (95% CI 10% to 20%)) in Gem. Effects were strongest in Kenya, but weakened in 2019, particularly among older AGYW. In uMkhanyakude, DREAMS invitees had greater self-efficacy compared with non-invitees in 2018 (+9% (95% CI 3% to 13%), 2018) but less so in 2019. In Kenyan settings, there was weak evidence for impact on self-efficacy among younger AGYW in Gem (+6% (95% CI 0% to 13%)) and older AGYW in Nairobi (+9% (95% CI −3% to +20%)) in 2019.

**Conclusions:**

DREAMS impacted on social support and, less consistently, on self-efficacy. Weakening effects over time may reflect changes in access to safe spaces and social networks as AGYW age and change circumstances, and withdrawal of DREAMS from uMkhanyakude in 2018, highlighting the importance of programme sustainability and improving programming for older participants.

**Key questions**

**What is already known?**

- Determined, Resilient, Empowered, AIDS-free, Mentored and Safe (DREAMS) is a multicomponent intervention that seeks to address the underlying causes of vulnerability to HIV infection, including by empowering adolescent girls and young women (AGYW).
- In theoretical frameworks developed to conceptualise women’s empowerment, there are three closely related dimensions: resources, agency and achievements. Agency is the ability to make and act on choices, it is enabled by access to resources, and achievements are the outcomes of people’s choices and efforts. In the process of empowerment, changes in one dimension can lead to changes in others.
- Agency may be enacted individually or collectively, and is likely to be facilitated by high self-efficacy as well as resources in the form of social support and social connectedness.
- There is some evidence that interventions have the potential to improve adolescents’ agency, although evidence from complex interventions implemented in ‘real-world’ settings is lacking.

**key questions**

**What are the new findings?**

- DREAMS increased social support among AGYW across diverse rural and urban settings in southern and eastern Africa, after 2–3 years of implementation.
- There was some impact of DREAMS on self-efficacy in the same time frame, with evidence of a positive impact in rural KwaZulu-Natal, South Africa and among younger AGYW in rural Kenya and older AGYW in Nairobi.

**What do the new findings imply?**

- Our findings support sustaining and expanding DREAMS, including safe spaces and mentoring, and suggest that holistic, multicomponent interventions can be implemented to improve aspects of AGYW’s empowerment.
- Enhancements to programming are needed for older AGYW, while increased engagement with communities is needed to ensure sustainability and adaptation to context.

## Background

Despite significant advances in HIV prevention, adolescent girls and young women (AGYW) aged 15–24 years are at considerably greater risk of HIV than their male peers throughout sub-Saharan Africa, accounting for one in four of all new HIV infections in the region in 2019.^1^ This is due, in part, to social and structural factors^1 2^ that perpetuate gender inequities and stifle the health and empowerment of young women. These social and structural factors include fewer years of schooling than male peers, food insecurity, engagement in ‘transactional’ sex for gifts or money, disparity in age with older male sexual partners, early marriage and gender-based violence.^2 3^

Women’s empowerment has been defined by Kabeer as ‘the processes by which those who have been denied the ability to make strategic life choices acquire such an ability’.^4^ Life choices include years of schooling, marriage, number of children, livelihoods, friends and networks, as well as choice around HIV prevention options including safer sex practices (eg, condom use, refusal of unwanted sex, pre-exposure prophylaxis).^4–6^ In this conceptual framing of empowerment, there are three dimensions and changes in one can lead to changes in others.^4 7^ A central dimension is agency, which describes ‘the ability to define one’s goals and act on them’ and may be exercised through reflection, decision making and negotiation.^4 7^ Women can exercise agency as individuals, and collectively with other women through formal and informal networks.^4 6 7^ A second dimension is resources, access to which can influence or determine what choices are made as well as how effectively they can be acted upon.^4 7^ The third dimension is achievements, which are the outcomes of people’s choices and also their efforts.^4 7^

Self-efficacy is described as a core property of human agency in social-cognitive theory,^8 9^ with one definition being ‘an optimistic sense of personal competence…accounting for motivation and accomplishments’.^10^ Individuals with high self-efficacy are thought to remain resilient in the face of adversity,^8^ to be able to initiate coping behaviour when needed,^11 12^ and to have belief in their ability to accomplish tasks, though this resilience and belief may not be sufficient to achieve a defined goal; the achievements that are possible may be limited by socio-structural factors, including societal norms and control exerted by partners and/or family members.^4 6 7 13^ The utility of measuring general self-efficacy, capturing a broad sense of personal competence, is widely acknowledged, though it can also be defined in relation to specific situations or domains, for example, condom use.^10 11 14–16^

In Kabeer’s framework, women’s empowerment is facilitated by, and may require, collective agency and solidarity.^4 7^ This is particularly the case in contexts where cultural norms may constrain women’s decision making and their ability to make their own life choices. For example, by standing together through mutual support and social networks, women may strengthen their voice, and gain greater control over their decisions and life choices.^4 6 7^ Social support, including social connectedness, is therefore an important element in increasing empowerment of AGYW.^17^ It can also be seen as a resource on which women may draw when making and acting on choices individually.

Together, self-efficacy, social support and social connectedness contribute to different dimensions of empowerment: ‘power within’ that drives individuals’ sense of agency and self-esteem, ‘power with’ other women that facilitates both individual and collective agency, and in combination the ‘power to’ make and act on decisions.^4 6 7^

The DREAMS (Determined, Resilient, Empowered, AIDS-free, Mentored and Safe) Partnership aims to reduce HIV incidence among AGYW through a holistic approach that addresses the complex underlying causes of vulnerability to HIV infection.^18^ The ‘core package’ includes evidence-based interventions that aim to enhance AGYW’s individual agency to access HIV prevention and sexual and reproductive health services.^19^ DREAMS also includes interventions to improve the social context in which AGYW live, for example, strengthening families of AGYW economically, enhancing parent-adolescent relationships, and mobilising communities, to elicit norms change. A fundamental component of the core package is social asset building, to strengthen both the individual and collective agency of AGYW (online supplemental file 1). Social asset building approaches enhance social networks of AGYW with female peers and mentors, through meetings in ‘safe spaces’, aiming to increase emotional and material support, resilience and self-esteem.^19^ Safe spaces typically refer to private, girl-only spaces established in, for example, community and church halls or schools, where AGYW can receive support and curriculum-based programmes, and be linked to other services. In a theory of change guiding analyses of DREAMS’ impact, these approaches are hypothesised to increase the agency of AGYW, and through this, contribute to reducing their vulnerability to HIV.^19^

10.1136/bmjgh-2021-006965.supp1 Supplementary data

While there is some evidence that interventions can increase adolescents’ agency, self-efficacy or social support, most previously reported studies were done under trial conditions, in specific settings such as schools and in high-income settings.^3 20–36^ For example, a career development curriculum for adolescent girls in high schools in the UA, including activities around self-awareness, decision making and gender identity, was reported to increase perceptions of social support and self-efficacy among other social cognitive and self-determination outcomes.^24^ In contrast, DREAMS was a complex intervention delivered at individual, family and community level, and in a ‘real-world’ context.

Here, we evaluate the impact of the combined DREAMS core package on social support and self-efficacy among population-based cohorts of AGYW in Kenya and South Africa, after 3 years of intervention delivery. We also sought to describe background levels of aspirations and expectations around important life milestones such as education and employment, to provide context to our findings.

## Methods

### Research settings

The research was carried out in three diverse settings, each capitalising on long-standing demographic surveillance platforms: in Kenya, the Nairobi Urban Health and Demographic Surveillance System (HDSS), established in 2002 in two informal settlements, and the Kenya Medical Research Institute/Centers for Disease Control and Prevention HDSS, established in 2001 in Gem, rural Siaya County; in South Africa, the Africa Health Research Institute HDSS, established in 1998 in rural, KwaZulu-Natal.^37–39^ The settings are characterised by a large youth population, and have historically high HIV prevalence and incidence.^40–44^

### DREAMS implementation context

Kenya and South Africa were identified by the US President’s Emergency Plan for AIDS Relief (PEPFAR) as priority countries for the implementation of DREAMS.^18^ Interventions were rolled out by DREAMS implementers from early 2016 in each country.^45^ Funding for DREAMS was stopped in uMkhanyakude in late 2018^46^ (because it was not among districts identified as ‘high-priority’ in the PEPFAR country operational plan) and continued in Kenya through 2019–2021. Models of delivery and ways of reaching AGYW in need varied by setting, described in detail previously.^45^ In South Africa, uMkhanyakude was selected following a geographical mapping exercise to identify DREAMS districts. AGYW were selected for DREAMS interventions by community-based organisations, from among the vulnerable children and families they worked with, and also through schools and social workers.^45 46^ In Kenyan settings, AGYW were invited to participate in DREAMS based on their risk characteristics such as being pregnant, or out-of-school or socioeconomically vulnerable, and were identified using the Girl Roster census method.^45 47^ The Girl Roster method enables rapid segmentation of AGYW into risk profile groups including those considered at particularly high risk, using a tool that collects information on age, marital status, childbearing, schooling and living arrangements. AGYW identified as vulnerable were invited to participate in DREAMS by implementing partners through door-to-door home visits followed by enrolment interviews.

### Evaluation study design and procedures

As part of an independent evaluation of the impact of DREAMS, described in detail previously,^48^ age-stratified, prospective, observational cohort studies of AGYW were conducted. AGYW aged 13–17 years (15–17 in Nairobi) and 18–22 years, residing in the HDSS area for each setting, were eligible and randomly selected for research cohort inclusion (therefore, capturing a random sample of those who had and had not been invited by implementing partners to participate in DREAMS interventions). Cohorts were enrolled in 2017 in Nairobi and uMkhanyakude, and 2018 in Gem, with annual follow-up until 2019; three rounds of data collection in total in Nairobi and uMkhanyakude and two rounds in Gem. At each round, participants were interviewed by trained data collectors to collect information on topics including sociodemographic and socioeconomic circumstances, sexual and pregnancy history, invitation to participate in DREAMS, self-efficacy beliefs and access to social support.

### Exposure measure

Our primary exposure measure was defined using self-reported data on invitation to participate in DREAMS (yes or no) that were collected using the research cohort study interview tool in all rounds of data collection. From this, we generated a binary variable that distinguished AGYW who were invited to DREAMS by 2018 from those who were not. Those invited to DREAMS by 2018 were considered DREAMS ‘beneficiaries’. ‘Non-beneficiaries’—those not invited by 2018—represent those who were not targeted or invited by implementing partners to participate in DREAMS interventions.

### Outcome measures

A binary, composite variable summarising social support, including social connectedness, was created using four questions on female networks and access to safe social spaces to meet^47 49^ (online supplemental file 2). A high level of social support was defined as a ‘yes’ response to three or more of the four questions, vs lower levels defined as ‘yes’ to between 0 and 2 questions. These decisions were guided by descriptive analyses for each setting that included the distribution of the number of ‘yes’ responses, overall and within age group strata and cross-tabulation of all pairs of component questions.

10.1136/bmjgh-2021-006965.supp2 Supplementary data

Ten questions comprising a general self-efficacy scale were used to create a binary self-efficacy outcome variable, measuring an overall coping ability, and competence to solve problems and meet goals^14^ (online supplemental file 3). The scale has been validated and used in numerous settings internationally.^10 50^ Responses to each scale question ranged from 1 (not at all true) to 4 (exactly true), with ‘not sure’ responses coded as zero (Nairobi only).^10^ Scores were summed across the 10 scale questions and an overall mean score calculated for each individual. Distributions were summarised and histograms plotted for visual inspection separately for each setting, overall, by age group, and by invitation to DREAMS. A cut-off value of ≥3.5, was used to define higher self-efficacy, with mean scores <3.5 indicating lower self-efficacy. This cut-off was selected as it lay, conceptually, between moderately and exactly true (scores of 3 and 4), was consistent with the literature,^51 52^ and was considered achievable, that is, a sizeable proportion of AGYW would fall into the higher self-efficacy category.

10.1136/bmjgh-2021-006965.supp3 Supplementary data

Questions on aspirations (phrased as ‘how important are the following things to you?’) and expectations (‘what are the chances that you will…?’) covered important life milestones such as education, employment, marriage and having children.

### Confounding factors

We constructed directed acyclic graphs (DAGs) using DAGitty^53 54^ to conceptualise and represent underlying causal structures, and identify a minimum set of confounders of the association between DREAMS exposure and each outcome, for inclusion in our statistical models. Factors potentially associated with the exposure and/or outcome were included in the DAGs based on local knowledge and related literature.

Confounding factors identified were measured at enrolment and included age group, geographic area or subsite, religion, ethnic group, educational attainment, currently attending school, socioeconomic status (wealth index), food insecurity, self-assessed household poverty, migration, sexual and pregnancy history, violence and orphanhood.

### Statistical analysis

Proportions reporting social support and self-efficacy in 2018 and 2019 were summarised overall, by age group, and by invitation to participate in DREAMS, separately for each setting. Results in 2018 were analysed among AGYW followed up in 2018, while results in 2019 were analysed among those followed up in 2019. Aspirations and expectations were also summarised by age group and invitation to DREAMS, for context.

We summarised associations between each characteristic at enrolment in 2017, guided by the minimal confounding set identified in the DAG, and invitation to DREAMS by 2018. We then conducted univariable logistic regression analyses for the association between each characteristic and the outcome. After adjusting for age and (for Nairobi and uMkhanyakude) area of residence a priori, we conducted multivariable logistic regression analyses of the effect of DREAMS invitation on social support/self-efficacy, adjusting for all characteristics in the minimum confounding set for each setting and outcome, plus those that were strong predictors of the outcome or considered potentially important in a particular context a priori (eg, migration for uMkhanyakude). Analyses were done overall, and separately for younger AGYW aged 13/15–17 years at cohort enrolment and older AGYW aged 18–22 years.

Next, using a causal inference framework, we estimated the causal effect of DREAMS on social support and self-efficacy by comparing the two counter-factual scenarios in which all, vs no, AGYW were DREAMS beneficiaries. Our primary analysis used propensity-score regression adjustment. The propensity score (PS)—the probability of being a DREAMS beneficiary given a set of characteristics—was predicted using a logistic regression model in which invitation to DREAMS by 2018 (yes/no) was specified as the ‘outcome’, and explanatory variables were confounders identified in the DAGs plus independent predictors of social support/self-efficacy. We then fitted a logistic regression model to predict the probability of social support/self-efficacy, restricted to AGYW who were DREAMS beneficiaries; age group and the PS were explanatory variables. From this model we predicted the probability of the outcome for *all* AGYW, irrespective of whether they were DREAMS beneficiaries. The average value of these probabilities was used to estimate the percentage of AGYW with the outcome under the counterfactual scenario that all AGYW were DREAMS beneficiaries. We repeated this approach for AGYW who were not DREAMS beneficiaries, to estimate the percentage of AGYW with the outcome under the counterfactual scenario that no AGYW were DREAMS beneficiaries. We present these average predictions overall, and separately for older and younger AGYW.

Sensitivity analyses were also done to check consistency of findings across different methodological approaches to control for confounding within the same framework (PS-stratification; PS-inverse-probability-of-treatment weighting; and using a multivariable logistic regression model of the outcome variable on the explanatory variables that were included in the PS model). We used bootstrapping on 1000 samples drawn with replacement to obtain confidence intervals for our predicted percentages with the outcome, and for the difference in the percentages between the two counterfactual scenarios for an absolute difference attributable to DREAMS.

### Patient and public involvement

Study findings were shared with the research participants and their communities, as well as health officials and programme implementers.

## Results

### Participants

In Nairobi, out of 1770 AGYW aged 15–22 years, residing in the study area and eligible to participate, 1081 (61%) were enrolled into a study cohort in 2017 (online supplemental file 4). Of these, 836 (77%) were followed up in 2018. In 2019, 117 AGYW not seen in 2018 were re-traced, while 101 dropped out, giving a total of 852 (79%) followed up at end-line. In Gem, out of 1258 eligible, 1171 were enrolled in 2018 (93%) and 1018 (87%) were followed up in 2019; in uMkhanyakude, 2527 were eligible, 2184 (86%) were enrolled in 2017, 1853 (85%) were followed up in 2018 and 1712 (78%) in 2019.

10.1136/bmjgh-2021-006965.supp4 Supplementary data

Patterns of loss to follow-up by participant characteristics at enrolment are presented in online supplemental file 5 and in detail elsewhere.^55^ Briefly, those not invited to participate in DREAMS, older, sexually active, out of school and food secure were less likely to be retained in the study.

10.1136/bmjgh-2021-006965.supp5 Supplementary data

Table 1 displays characteristics at cohort enrolment of participants followed up in 2019. Across the three settings, slightly higher proportions of AGYW aged 13/15–17 were enrolled than older AGYW aged 18–22. Proportions reporting food insecurity ranged from 23% to 34%. Most adolescents aged <18 years had never had sex, and among older AGYW, over 60% were sexually active and over 30% had been pregnant (online supplemental file 6). Proportions in school were high, particularly among the younger cohorts, and most AGYW aged ≥18 had progressed to secondary education. The overall proportions invited to participate in DREAMS by 2018 were 57% in Gem, 74% in Nairobi and 53% in uMkhanyakude. Higher proportions of those invited to DREAMS by 2018 were younger, in school, never had sex, food insecure and from lower SES households, compared with those never invited. Further details, including factors independently associated with invitation to participate in DREAMS, are published elsewhere.^55 56^

10.1136/bmjgh-2021-006965.supp6 Supplementary data

**Table 1:** Sociodemographic characteristics of DREAMS beneficiaries and non-beneficiaries at the time of cohort enrolment in Gem (2018), Nairobi and uMkhanyakude (2017), among those followed up in 2019

Characteristics at enrolment	Gem			Nairobi			uMkhanyakude		
	Overall (N=1018)	Never invited (N=436)	Invited in 2018 (N=582)	Overall (N=852)	Never invited (N=224)	Invited by 2018 (N=628)	Overall (N=1712)	Never invited (N=809)	Invited by 2018 (N=903)
	% (col)	% (col)	% (col)	% (col)	% (col)	% (col)	% (col)	% (col)	% (col)
Age									
13/15–17	61.1	59.9	62.0	54.5	42.4	58.8	56.8	45.0	67.3
18–22	38.9	40.1	38.0	45.5	57.6	41.2	43.2	55.0	32.7
Currently in school									
No				36.6	48.7	32.3	21.0	30.7	12.3
Yes				63.4	51.3	67.7	79.0	69.3	87.7
Education completed									
None/primary	42.7	40.1	44.7						
Secondary/tertiary	36.5	32.8	39.3						
Unknown	20.7	27.1	16.0						
Education completed									
None/some primary				10.8	13.4	9.9	10.3	8.3	12.1
Primary/some secondary				68.1	58.0	71.7	77.3	73.1	81.1
Secondary/tertiary				21.1	28.6	18.5	12.4	18.6	6.8
Food insecure									
No	77.5	82.6	73.7	66.2	74.1	63.4	68.8	65.4	71.9
Yes	22.5	17.4	26.3	33.8	25.9	36.6	31.2	34.6	28.1
Socioeconomic status									
Low	41.7	36.0	45.9	35.6	34.4	36.0	35.9	32.2	39.3
Medium	19.2	19.0	19.2	32.5	35.3	31.5	35.0	36.1	34.0
High	39.2	45.0	34.9	31.9	30.4	32.5	29.1	31.8	26.7
Ever had sex									
No	68.9	64.0	72.5	65.4	55.8	68.8	63.4	54.2	71.4
Yes	31.1	36.0	27.5	34.6	44.2	31.2	36.7	45.8	28.6
Ever pregnant									
No	84.4	81.4	86.6	75.9	67.4	79.0	75.2	67.8	81.8
yes	15.6	18.6	13.4	24.1	32.6	21.0	24.8	32.2	18.2

DREAMS, Determined, Resilient, Empowered, AIDS-free, Mentored and Safe.

### Descriptive summary of aspirations and expectations

Aspirations around education, employment and home ownership were high (across settings in 2019,≥88% considered important for each statement), with few differences by age group, DREAMS invitation status or year (online supplemental file 7). An exception was educational aspirations in Kenya where among younger AGYW in Nairobi in 2019, 94% of DREAMS-invitees thought finishing secondary school was very important vs 86% of non-invitees, and among older AGYW in Gem, 89% of DREAMS invitees thought accessing tertiary education was very important vs 81% of non-invitees. The majority considered children and marriage/partnerships as important, with a much higher proportion in Kenya (eg, Nairobi: 95% and 88%, respectively) than in uMkhanyakude (56% and 57%), and more among AGYW aged ≥18 vs younger adolescents (eg, Gem: 85% vs 74% for having children).

10.1136/bmjgh-2021-006965.supp7 Supplementary data

Expectations around similar life milestones were slightly lower than aspirations (online supplemental file 7). In both Kenyan settings and among older AGYW in uMkhanyakude, higher expectations were reported for education, employment and health-related expectations among AGYW invited to DREAMS versus those never invited, though differences were modest, for example, within ±5% in absolute terms, for most statements.

### Patterns of social support by setting, year, age and DREAMS exposure

Levels of social support were highest in Nairobi (56% overall, 2019) and lowest in Gem (40%), with a small increase from 2018 to 2019 in both Kenyan settings and no change in uMkhanyakude (table 2). In all settings, both age groups, and in both years of follow-up, proportions with high social support were greater among DREAMS beneficiaries versus non-beneficiaries. For example, in Gem in 2018 the percentage of younger AGYW with social support was 39% among DREAMS beneficiaries vs 27% among non-beneficiaries. Comparing responses for the component questions comprising our social support measure, the greatest differences between beneficiaries and non-beneficiaries were for having a ‘safe and private place to meet’, particularly in Kenyan settings (eg, 59% vs 40%, Nairobi, 2019) (online supplemental file 2).

**Table 2:** Crude and multivariable analyses for associations between invitation to participate in DREAMS and outcomes (social support; self-efficacy) in 2018/2019

Outcome	Setting	Age group	All		Never invited		Invited by 2018		Unadjusted OR (95% CI)	Age and area adjusted OR (95% CI)	Fully adjusted* OR (95% CI)	P value (LRT)
			N	n (%) with outcome	N	n (%) with outcome	N	n (%) with outcome				
Social support, 2018	Nairobi	Overall	831	421 (50.7)	210	90 (42.9)	621	331 (53.3)	1.5 (1.1 to 2.1)	1.5 (1.1 to 2.1)	1.5 (1.1 to 2.1)	0.01
		15–17	466	240 (51.5)	94	33 (35.1)	372	207 (55.7)	2.3 (1.4 to 3.7)	2.2 (1.4 to 3.5)	2.4 (1.5 to 3.9)	<0.001
		18–22	365	181 (49.6)	116	57 (49.1)	249	124 (49.8)	1.0 (0.7 to 1.6)	1.1 (0.7 to 1.7)	1.0 (0.6 to 1.7)	0.9
	Gem	Overall	1171	424 (36.2)	514	145 (28.2)	657	279 (42.5)	1.9 (1.5 to 2.4)	2.0 (1.5 to 2.5)	2.0 (1.6 to 2.6)	<0.001
		13–17	684	231 (33.8)	285	76 (26.7)	399	155 (38.8)	1.8 (1.3 to 2.4)	1.8 (1.3 to 2.5)	2.0 (1.4 to 2.8)	<0.001
		18–22	487	193 (39.6)	229	69 (30.1)	258	124 (48.1)	2.2 (1.5 to 3.1)	2.3 (1.6 to 3.4)	2.4 (1.6 to 3.6)	<0.001
	uMkhanyakude	Overall	1852	847 (45.7)	886	373 (42.1)	966	474 (49.1)	1.3 (1.1 to 1.6)	1.4 (1.1 to 1.6)	1.4 (1.1 to 1.7)	0.002
		13–17	1040	490 (47.1)	389	170 (43.7)	651	320 (49.2)	1.2 (1.0 to 1.6)	1.3 (1.0 to 1.7)	1.3 (1.0 to 1.7)	0.03
		18–22	812	357 (44.0)	497	203 (40.9)	315	154 (48.9)	1.4 (1.0 to 1.8)	1.4 (1.1 to 1.9)	1.4 (1.1 to 1.9)	0.02
Social support, 2019	Nairobi	Overall	852	480 (56.3)	224	111 (49.6)	628	369 (58.8)	1.4 (1.1 to 2.0)	1.4 (1.0 to 1.9)	1.4 (1.0 to 1.9)	0.04
		15–17	464	266 (57.3)	95	43 (45.3)	369	223 (60.4)	1.8 (1.2 to 2.9)	1.7 (1.1 to 2.8)	1.7 (1.0 to 2.8)	0.03
		18–22	388	214 (55.2)	129	68 (52.7)	259	146 (56.4)	1.2 (0.8 to 1.8)	1.2 (0.8 to 1.8)	1.2 (0.8 to 1.9)	0.4
	Gem	Overall	1018	411 (40.1)	436	156 (35.8)	582	255 (43.8)	1.4 (1.1 to 1.8)	1.4 (1.1 to 1.8)	1.4 (1.0 to 1.8)	0.02
		13–17	622	229 (37.0)	261	81 (31.0)	361	148 (41.0)	1.5 (1.1 to 2.2)	1.5 (1.1 to 2.2)	1.6 (1.1 to 2.3)	0.008
		18–22	396	182 (46.0)	175	75 (42.9)	221	107 (48.4)	1.3 (0.8 to 1.9)	1.3 (0.8 to 1.9)	1.1 (0.7 to 1.7)	0.6
	uMkhanyakude	Overall	1712	778 (45.4)	809	358 (44.3)	903	420 (46.5)	1.1 (0.9 to 1.3)	1.1 (0.9 to 1.3)	1.1 (0.9 to 1.3)	0.5
		13–17	972	441 (45.4)	364	157 (43.1)	608	284 (46.7)	1.2 (0.9 to 1.5)	1.1 (0.9 to 1.5)	1.2 (0.9 to 1.5)	0.2
		18–22	740	337 (45.5)	445	201 (45.2)	295	136 (46.1)	1.0 (0.8 to 1.4)	1.0 (0.8 to 1.4)	0.9 (0.7 to 1.3)	0.7
Self efficacy, 2018	Nairobi	Overall	836	449 (53.7)	212	109 (51.4)	624	340 (54.5)	1.1 (0.8 to 1.6)	1.2 (0.8 to 1.6)	1.1 (0.8 to 1.6)	0.5
		15–17	466	243 (52.1)	94	49 (52.1)	372	194 (52.2)	1.0 (0.6 to 1.6)	1.0 (0.6 to 1.6)	1.0 (0.6 to 1.6)	0.9
		18–22	370	206 (55.7)	118	60 (50.8)	252	146 (57.9)	1.3 (0.9 to 2.1)	1.4 (0.9 to 2.1)	1.2 (0.8 to 2.0)	0.4
	Gem	Overall	1171	436 (37.2)	514	193 (37.5)	657	243 (37.0)	1.0 (0.8 to 1.2)	1.0 (0.8 to 1.3)	1.1 (0.9 to 1.5)	0.4
		15–17	684	224 (32.7)	285	93 (32.6)	399	131 (32.8)	1.0 (0.7 to 1.4)	1.0 (0.7 to 1.4)	1.1 (0.8 to 1.5)	0.6
		18–22	487	212 (43.5)	229	100 (43.7)	258	112 (43.4)	1.0 (0.7 to 1.4)	1.0 (0.7 to 1.4)	1.1 (0.7 to 1.6)	0.7
	uMkhanyakude	Overall	1853	771 (41.6)	886	348 (39.3)	967	423 (43.7)	1.2 (1.0 to 1.4)	1.4 (1.1 to 1.7)	1.4 (1.2 to 1.8)	<0.001
		15–17	1041	379 (36.4)	389	125 (32.1)	652	254 (39.0)	1.3 (1.0 to 1.8)	1.3 (1.0 to 1.7)	1.4 (1.0 to 1.8)	0.03
		18–22	812	392 (48.3)	497	223 (44.9)	315	169 (53.7)	1.4 (1.1 to 1.9)	1.5 (1.1 to 2.0)	1.5 (1.1 to 2.1)	0.004
Self efficacy, 2019	Nairobi	Overall	852	465 (54.6)	224	113 (50.5)	628	352 (56.1)	1.3 (0.9 to 1.7)	1.3 (0.9 to 1.8)	1.3 (0.9 to 1.8)	0.1
		15–17	464	247 (53.2)	95	48 (50.5)	369	199 (54.0)	1.2 (0.7 to 1.8)	1.1 (0.7 to 1.7)	1.1 (0.7 to 1.7)	0.8
		18–22	388	218 (56.2)	129	65 (50.4)	259	153 (59.1)	1.4 (0.9 to 2.2)	1.4 (0.9 to 2.2)	1.6 (1.0 to 2.6)	0.04
	Gem	Overall	1018	351 (34.5)	436	145 (33.3)	582	206 (35.4)	1.1 (0.9 to 1.4)	1.1 (0.9 to 1.5)	1.2 (0.9 to 1.5)	0.2
		15–17	622	187 (30.1)	261	71 (27.2)	361	116 (32.1)	1.3 (0.9 to 1.8)	1.3 (0.9 to 1.8)	1.5 (1.0 to 2.2)	0.04
		18–22	396	164 (41.4)	175	74 (42.3)	221	90 (40.7)	0.9 (0.6 to 1.4)	0.9 (0.6 to 1.4)	1.0 (0.6 to 1.5)	0.8
	uMkhanyakude	Overall	1712	829 (48.4)	809	384 (47.5)	903	445 (49.3)	1.1 (0.9 to 1.3)	1.2 (1.0 to 1.5)	1.3 (1.0 to 1.5)	0.03
		15–17	972	414 (42.6)	364	140 (38.5)	608	274 (45.1)	1.3 (1.0 to 1.7)	1.3 (1.0 to 1.7)	1.3 (1.0 to 1.7)	0.04
		18–22	740	415 (56.1)	445	244 (54.8)	295	171 (58.0)	1.1 (0.8 to 1.5)	1.1 (0.8 to 1.6)	1.2 (0.8 to 1.6)	0.4

Row percentages are presented.

Outcome definition for social support: Binary outcome variable constructed where a high level of social support was defined as a ‘yes’ response to at least three out of four questions: ‘Is there a female in your community from whom you can borrow money in an emergency?’; ‘Do you have at least one trusted female friend?’; ‘Do you know a woman in your community, other than a mother or guardian, whom you could turn to if you had a serious problem?’; ‘Do you have a safe and private place to meet with girls and young women who are like you?’ Outcome definition for self-efficacy: Binary outcome variable constructed based on a series of 10 questions comprising a general self-efficacy scale, where a cut-off value of ≥3.5 was used to define higher self-efficacy (yes).

*Adjusted for the following variables:Gem: Social support: age group (categorised as 13–17/18–22), education (none or primary/secondary and above/ unknown), socioeconomic status (wealth index derived using principal component analysis with input variables including, for example, individual or household assets and household structure; categorised as low/medium/high), orphanhood (no/maternal/paternal/double orphan/unknown based on self-reports of mother or father having died), food insecurity (AGYW or household member went to sleep at night hungry because there was not enough food in the past 4 weeks; yes/no) and sexual and pregnancy history (never had sex/had sex never pregnant/ever pregnant); Self-efficacy: age group, education, socioeconomic status, orphanhood, food insecurity and sexual and pregnancy history (variable definitions the same as for social support analyses).Nairobi: Social support: age group and education (composite variable combining age 15–17/18–22, in school/not in school, no/primary/secondary/tertiary education attainment), Demographic Surveillance System (DSS) study site (Korogocho/Viwandani settlements), marital status (never married/previously married or living with partner/currently married or living with partner), sexual and pregnancy history (never had sex/had sex never pregnant/ever pregnant), socioeconomic status (wealth index derived using principal component analysis with input variables including for example, individual or household assets and household structure; categorised as low/medium/high), food insecurity (AGYW or household member went to sleep at night hungry because there was not enough food in the past 4 weeks; yes/no), poverty perception (self-assessment of household economic situation currently as very poor/moderately poor/not poor), orphanhood (single or double orphan/ not orphan, based on self-reports of mother or father having died); Self-efficacy: DSS study site, food insecurity, orphanhood, sexual and pregnancy history, socioeconomic status, poverty perception (variable definitions the same as for social support analyses), age group (15–17/18–22), in/out of school (yes/no), birth history (ever given birth yes/no), ethnic group (Somali, Kamba, Kikuyu, Kisii, Luhya, Luo, other), religion (Catholic/other Christian/Muslim/no or other religion), gender of the household head (male/female), AGYW was the household head (yes/no).uMkhanyakude: Social support: age group (13–14/15–17/18–19/20–22), age and education (composite and dummy variables comparing age groups 13–17/18–22, in school/not in school, incomplete/complete secondary education), area (rural/ periurban or urban), sexual and pregnancy history (never had sex/had sex never pregnant/ever pregnant), socioeconomic status (wealth index derived using principal component analysis with input variables including for example, individual or household assets and household structure; categorised as low/medium/high), food insecurity (any report of reducing the size of food portions or skipping meals by any member of a household because there was not enough money to buy food in the past 12 months), migration (any movement within or outside the surveillance area since age 13); Self-efficacy: age group, age and education, area, sexual and pregnancy history, socioeconomic status, food insecurity, migration (variable definitions the same as for social support analyses), violence (experience of any act of violence by a man in the 12 months preceding the survey).

AGYW, adolescent girls and young women; DREAMS, Determined, Resilient, Empowered, AIDS-free, Mentored and Safe; LRT, likelihood ratio test; OR, odds ratio.

### Estimated impact of DREAMS on social support

The odds of having high social support in 2018 were greater among DREAMS beneficiaries vs non-beneficiaries in all settings (eg, adjusted OR (aOR) 1.5 (95%CI 1.1 to 2.1), Nairobi) (table 2; online supplemental file 8), in younger AGYW, and in older AGYW in Gem and uMkhanyakude. In 2019, evidence for an association with DREAMS weakened, particularly in uMkhanyakude (aOR 1.1 (95% CI 0.9 to 1.3) overall) and in the older cohorts of AGYW (eg, aOR 1.1 (95% CI 0.7 to 1.7), Gem). However, in Kenya, evidence remained for greater odds of social support in 2019 among DREAMS beneficiaries vs non-beneficiaries overall (eg, aOR 1.4 (95% CI 1.0 to 1.8), p=0.02, Gem) and in the younger cohorts (eg, aOR 1.7 (95% CI 1.0 to 2.8), p=0.03, Nairobi).

10.1136/bmjgh-2021-006965.supp8 Supplementary data

**Figure 1:**
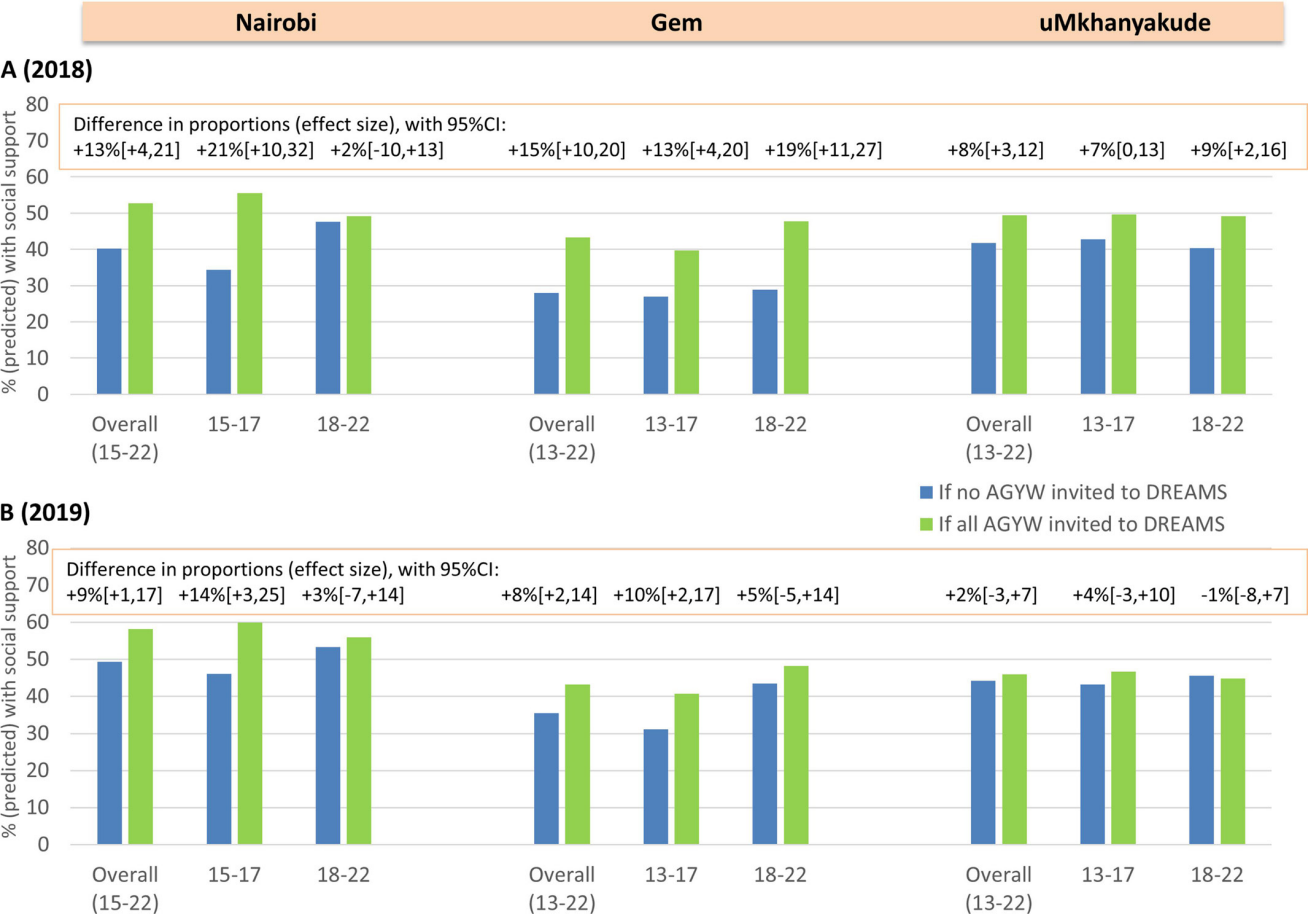
Predicted proportions who have social support in 2018 (A) and in 2019 (B) if no AGYW versus all AGYW were invited to DREAMS, overall and by age group at enrolment in three settings. AGYW, adolescent girls and young women; DREAMS, Determined, Resilient, Empowered, AIDS-free, Mentored and Safe.

In 2018, we estimated that the percentage of AGYW with social support would increase from 28% if none were DREAMS beneficiaries to 43% if all were beneficiaries (+15% (95%CI +10% to 20%)) in Gem, with corresponding figures of 40% and 53% in Nairobi (+13% (95% CI +4% to 21%)) and 42% and 49% in uMkhanyakude (+8% (95% CI +3% to 12%)) (table 3, figure 1). Increases were estimated among younger AGYW in all settings, and among older AGYW in Gem and uMkhanyakude, with the exception being older AGYW in Nairobi where there was no evidence for a difference in predicted percentages with social support between the scenarios that no, vs all AGYW were DREAMS beneficiaries (+2% (95% CI −10% to +13%)). Differences attributable to DREAMS were largest in Kenya (eg, +21% (95% CI +10% to 32%) among 15–17 year-olds, Nairobi, 2018), and weakened in 2019, particularly among older AGYW (eg, +5% (95% CI −5% to +14%), Gem) and overall in uMkhanyakude (+2% (95% CI −3% to +7%)). Results were similar in sensitivity analyses that used alternative approaches to control for confounding (online supplemental file 9).

10.1136/bmjgh-2021-006965.supp9 Supplementary data

**Table 3:** Estimated causal effect of DREAMS on social support and self-efficacy in 2018 and in 2019

Outcome and year	Setting	Age group	% with outcome in total study population	Estimated % with outcome if none benefit from DREAMS	Estimated % with outcome if all benefit from DREAMS	Difference in estimated %
				% (95% CI)	% (95% CI)	% (95% CI)
Social support, 2018	Nairobi	Overall	50.7	40.2 (33.1 to 47.6)	52.7 (48.5 to 56.7)	12.5 (4.2 to 20.9)
		15–17	51.5	34.4 (24.8 to 44.3)	55.5 (50.4 to 60.8)	21.1 (9.6 to 31.8)
		18–22	49.6	47.6 (38.4 to 56.6)	49.2 (42.8 to 55.6)	1.6 (-10.1 to 13.3)
	Gem	Overall	36.2	27.9 (23.9 to 32.9)	43.3 (39.6 to 46.8)	15.4 (10.2 to 19.8)
		13–17	33.8	26.9 (22.0 to 35.2)	39.7 (35.4 to 43.6)	12.8 (4.0 to 19.8)
		18–22	39.6	28.8 (22.5 to 35.5)	47.8 (42.9 to 53.4)	19.0 (10.6 to 27.1)
	uMkhanyakude	Overall	45.7	41.7 (38.4 to 45.4)	49.4 (46.1 to 52.8)	7.7 (2.5 to 12.0)
		13–17	47.1	42.8 (38.2 to 47.9)	49.6 (45.6 to 53.3)	6.8 (-0.3 to 12.9)
		18–22	44.0	40.4 (35.9 to 45.1)	49.2 (43.2 to 55.0)	8.8 (1.6 to 15.8)
Social support, 2019	Nairobi	Overall	56.3	49.4 (42.6 to 56.2)	58.2 (54.1 to 62.3)	8.8 (1.2 to 16.7)
		15–17	57.3	46.1 (36.2 to 55.6)	60.0 (54.6 to 65.0)	14.0 (3.0 to 25.0)
		18–22	55.2	53.3 (43.9 to 61.3)	56.0 (49.5 to 62.5)	2.6 (-7.3 to 14.1)
	Gem	Overall	40.4	35.5 (31.1 to 39.6)	43.3 (39.3 to 47.3)	7.8 (2.1 to 14.0)
		13–17	37.0	31.2 (26.0 to 37.3)	40.7 (35.7 to 46.3)	9.5 (1.9 to 17.3)
		18–22	44.9	43.5 (35.7 to 51.1)	48.2 (41.6 to 55.0)	4.7 (-4.5 to 14.1)
	uMkhanyakude	Overall	45.4	44.2 (40.5 to 47.7)	46.0 (42.5 to 49.5)	1.8 (-3.2 to 6.6)
		13–17	45.4	43.2 (38.0 to 48.1)	46.7 (42.7 to 50.7)	3.6 (-2.6 to 10.2)
		18–22	45.5	45.6 (41.2 to 50.4)	44.9 (39.1 to 50.4)	−0.6 (-7.5 to 6.6)
Self-efficacy, 2018	Nairobi	Overall	53.7	54.7 (46.4 to 60.7)	54.9 (51.2 to 59.2)	1.2 (-6.9 to 10.0)
		15–17	52.1	53.7 (43.3 to 64.5)	52.1 (47.4 to 57.3)	−1.6 (-13.7 to 9.9)
		18–22	55.7	53.6 (43.8 to 63.2)	58.4 (52.8 to 64.5)	4.8 (-6.4 to 16.6)
	Gem	Overall	37.2	36.1 (30.9 to 40.6)	38.5 (35.3 to 41.8)	2.4 (-4.1 to 8.5)
		13–17	32.8	32.7 (27.1 to 37.7)	34.7 (29.8 to 38.9)	2.0 (-4.3 to 9.6)
		18–22	43.5	40.3 (34.5 to 46.0)	43.4 (36.7 to 48.9)	3.2 (-3.9 to 9.7)
	uMkhanyakude	Overall	41.6	37.9 (34.5 to 41.4)	46.4 (42.9 to 49.6)	8.6 (3.4 to 13.1)
		13–17	36.4	32.4 (27.6 to 37.3)	39.4 (35.6 to 43.3)	7.0 (0.9 to 12.9)
		18–22	48.3	44.9 (40.6 to 49.8)	55.5 (50.0 to 61.4)	10.6 (3.2 to 17.8)
Self-efficacy, 2019	Nairobi	Overall	54.6	50.5 (43.8 to 58.2)	56.3 (52.2 to 60.2)	5.7 (-2.7 to 13.9)
		15–17	53.2	50.6 (41.3 to 61.2)	53.9 (49.0 to 59.0)	3.3 (-8.7 to 14.3)
		18–22	56.2	50.5 (41.9 to 59.5)	59.1 (53.2 to 65.5)	8.6 (-3.0 to 19.8)
	Gem	Overall	34.5	31.8 (27.3 to 36.0)	35.6 (31.1 to 39.0)	3.8 (-4.0 to 9.1)
		13–17	30.1	27 (21.6 to 31.7)	32.8 (28.2 to 37.8)	5.7 (-0.1 to 13.4)
		18–22	41.4	41 (33.6 to 49.8)	41.2 (34.2 to 46.6)	0.2 (-11.7 to 9.6)
	uMkhanyakude	Overall	48.4	45.5 (42.1 to 49.3)	51.0 (47.4 to 54.4)	5.4 (0.5 to 10.1)
		13–17	42.6	38.7 (33.6 to 44.1)	45.3 (41.4 to 49.7)	6.7 (0.3 to 12.6)
		18–22	56.1	54.5 (49.9 to 59.4)	58.3 (52.2 to 64.0)	3.8 (-3.6 to 11.2)

2018 denominator: AGYW followed up in 2018 (Overall totals: Gem 1171; Nairobi 836; uMkhanyakude 1853).

2019 denominator: AGYW followed up in 2019 (Overall totals: Gem 1018; Nairobi 852; uMkhanyakude 1712).

Method: Propensity-score regression adjustment.

Outcome definition social support: Binary outcome variable constructed where a high level of social support was defined as a ‘yes’ response to at least three out of four questions: ‘Is there a female in your community from whom you can borrow money in an emergency?’; ‘Do you have at least one trusted female friend?’; ‘Do you know a woman in your community, other than a mother or guardian, whom you could turn to if you had a serious problem?’; ‘Do you have a safe and private place to meet with girls and young women who are like you?’Outcome definition self-efficacy: Binary outcome variable constructed based on a series of ten questions comprising a general self-efficacy scale, where a cut-off value of ≥3.5 was used to define higher self-efficacy (yes).

AGYW, adolescent girls and young women; DREAMS, Determined, Resilient, Empowered, AIDS-free, Mentored and Safe.

### Patterns of self-efficacy by setting, year, age and DREAMS exposure

Proportions with high self-efficacy were greater in Nairobi (eg, 54%, 2018) than in uMkhanyakude (42%, 2018) or Gem (37%, 2018), and higher among older versus younger AGYW (eg, 41% vs 30% in Gem, 2019) (table 2, online supplemental file 3). Levels did not change by 2019 in Kenyan settings, although self-efficacy rose to 48% in uMkhanyakude. Overall, proportions with high self-efficacy were similar or slightly greater among those invited to DREAMS compared with those never invited, with greater differences by subgroups of age, for example, 59% vs 50% among older AGYW in Nairobi in 2019.

### Estimated impact of DREAMS on self-efficacy

Overall, there was no evidence for an effect of DREAMS on self-efficacy in Kenyan settings in either year (eg, aOR 1.2 (95% CI 0.9 to 1.5), Gem, 2019) (table 2; online supplemental file 8). However, a modest effect was observed in 2019 among younger AGYW in Gem (aOR 1.5 (95% CI 1.0 to 2.2)) and older AGYW in Nairobi (aOR 1.6 (95% CI 1.0 to 2.6)). In uMkhanyakude, DREAMS beneficiaries had greater odds of high self-efficacy compared with non-beneficiaries overall (aOR 1.4 (95% CI 1.2 to 1.8), 2018; 1.3 (1.0 to 1.5), 2019), and aORs were similar in subgroup analyses by age group.

**Figure 2:**
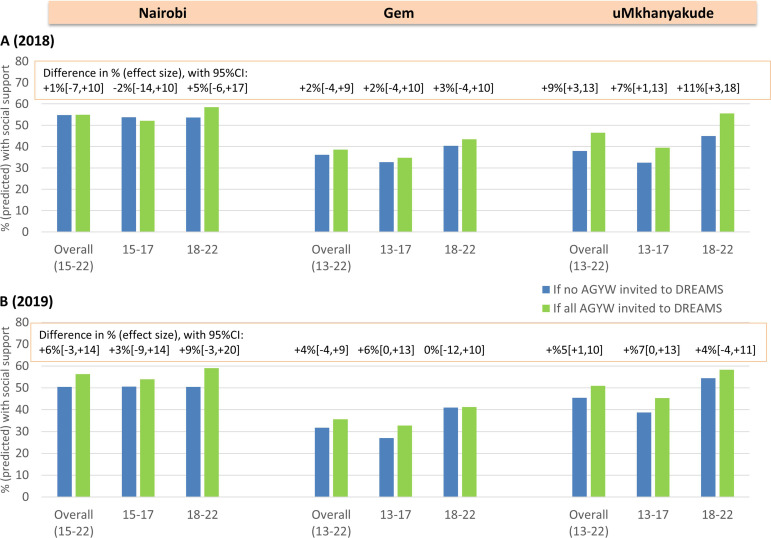
Predicted proportions who have self efficacy in 2018 (A) and in 2019 (B) if no AGYW versus all AGYW were invited to DREAMS, overall and by age group at enrolment in three settings. AGYW, adolescent girls and young women; DREAMS, Determined, Resilient, Empowered, AIDS-free, Mentored and Safe.

In uMkhanyakude, we estimated that DREAMS would increase self-efficacy in 2018 from 38% if no AGYW were DREAMS beneficiaries to 46% if all AGYW were beneficiaries (+9% (95% CI +3% to 13%)). The predicted increase was slightly weaker in 2019, particularly among older AGYW (+4% (95% CI −4% to +11%)) (table 3, figure 2). In Kenyan settings, there was no evidence for an effect of DREAMS in 2018, while in 2019 there was weak evidence for a positive impact of DREAMS among younger AGYW in Gem (+6% (95% CI 0% to 13%)) and among older AGYW in Nairobi (+9% (95% CI −3% to +20%). Results were similar in sensitivity analyses (online supplemental file 9).

## Discussion

### Key findings

DREAMS increased social support among AGYW across diverse rural and urban settings in southern and eastern Africa. We also found some impact of DREAMS on self-efficacy, with evidence of a positive impact in rural KwaZulu-Natal, and among younger AGYW in rural Kenya and older AGYW in Nairobi. Aspirations and expectations were high, and there were examples of modestly elevated expectations for education, employment and health-related milestones among DREAMS beneficiaries compared with non-beneficiaries.

### Interpretation of social support findings

The DREAMS package aimed to create an enabling environment through interventions that strengthen families and elicit community-wide norms change. Social asset building approaches were specifically included to strengthen networks of AGYW with peers and female mentors, helping AGYW to feel socially supported with a collective and connected identity.^4 6 7 19^ We previously reported good uptake of the DREAMS package over the same time frame (2017–2019), with almost all AGYW invited to DREAMS participating in at least one intervention, and many accessing multiple (eg, 3+) interventions.^55–57^ Social asset building interventions in particular, including safe spaces,^19 55–57^ were highly accessed (particularly by younger AGYW), so the observed impacts of DREAMS on social support are plausible from an implementation perspective, and could reflect exposure to valuable social resources as conceptualised in Kabeer’s empowerment framework.^4^ As our definition of social support captured access to a safe and private place, as well as connectedness and support from other females, it is likely that the impacts due to DREAMS primarily reflect participation in social asset building interventions, and to a lesser extent participation in DREAMS school-based and social protection curricula which may also have enhanced opportunities for social networking.

### Interpretation of self-efficacy findings

The enabling, supportive environment created through the DREAMS package of interventions was also hypothesised to boost individual agency and general self-efficacy, facilitating decisions around access to HIV prevention and sexual and reproductive health services including testing, condoms and family planning. While impacts of DREAMS on social support may occur relatively quickly, it may take longer and more sustained intervention^45^ to achieve impacts on self-efficacy beliefs. This is one possible explanation for the relatively weak effects of DREAMS on self-efficacy by 2019, and for the heterogeneity across settings. Longer-term follow-up, after interventions have become embedded and then sustained with sufficient intensity, might show a larger change in attitudes and beliefs.

Broader societal influences, including poverty, economic circumstances, family, male partners and cultural norms, are also thought to affect what choices are considered possible and the extent to which choice can be exercised.^4 6 7 13 45 58^ These wider issues may have limited the impact of DREAMS interventions on the individual agency of AGYW and their self-efficacy beliefs. For instance, uptake of DREAMS community norms-change interventions was low in the general populations in our study settings,^56^ and DREAMS may not have influenced these broader contextual factors very much. Another reason for the modest levels of self-efficacy observed and weak effects of DREAMS could be the fairly stringent cut-off used to define self-efficacy.

There may be differences between Kenya and South Africa, and between settings in Kenya, in AGYW’s perceptions around access to resources (including HIV prevention tools), which will in turn influence their perceived choices and decision-making. This may offer another explanation for the heterogeneity in self-efficacy findings, and further qualitative research would be valuable for better understanding.

### Findings in context

Impacts of DREAMS specifically on social support and general self-efficacy have not been reported elsewhere. However, complementing our findings, implementation science research conducted in Zambia and Kenya found that high proportions of DREAMS beneficiaries felt comfortable with their mentors and that mentors were ‘readily available when an issue arose’.^59 60^ The impacts seen in our study support the continued expansion of safe social spaces where AGYW can meet, engage in transformative communications and learning, and initiate collective action, through peer-networking and peer mentorship, as part of a holistic approach to combination HIV prevention.^61–64^

Cohort studies with DREAMS beneficiaries in Zambia and Kenya reported high levels of self-efficacy for HIV testing, and self-perceptions of reduced HIV risk,^59 60^ but the absence of a comparison group of non-beneficiaries in the research hinders interpretation of impact. Several Africa-based studies assessing educational, health promotion or economic empowerment interventions have also reported positive effects on specific forms of self-efficacy, though these findings were generally from trial contexts or pre-/post-intervention comparisons that may be confounded by other contributing factors.^32–34 36^ For example, a cohort study with young people living with HIV in Uganda who participated in a peer-led intervention package of HIV and sexual and reproductive health services reported increases in self-efficacy ‘to engage in healthy behaviours’ after 9 months of the intervention.^31^ Our study, therefore, makes an important contribution to understanding whether complex interventions can be implemented to impact on self-efficacy among young people in real-world contexts.

### Impacts by age group

On the whole, stronger impacts on social support and self-efficacy were seen among younger vs older AGYW. We also observed that uptake of relevant interventions, including social asset building, and ‘layering’ of interventions across the DREAMS core package, were generally greater in this age group.^31 55–57^ Weaker impacts among older AGYW may also reflect challenges engaging them in the programme over a sustained period, for example, due to competing priorities to care for family, or short-term migration to earn a living, and consequently less freedom and choice about how to spend their time.^45 46^ Completion of curricula or programme disengagement are also possible explanations for weakening effects of DREAMS in 2019 among the older cohorts, as well as ageing of the cohorts, again indicating that adaptation and/or new ways to sustain social support would be valuable as AGYW age and their life circumstances (including relationships and marriage) evolve. Involving older AGYW in the adaptation and refinement of DREAMS interventions will be essential to ensure that curricula are useful and stimulating and offered in a way that is compatible with competing demands on their time, so as to contribute to strengthening their social networks and support, self-efficacy beliefs and ultimately their agency.

### Impacts over time

Impacts on social support were weaker in 2019 than in 2018 across all settings, particularly in uMkhanyakude, where impacts on self-efficacy also weakened over time. In uMkhanyakude, this likely reflects the withdrawal of DREAMS funding in late 2018, and corresponding evidence of weakening participation in DREAMS interventions, particularly social asset building.^46 55 57^ This emphasises the importance of sustainability, including ongoing support for safe social spaces and continued opportunities for communication with mentors and/or peer-networks and of further engaging communities in leadership.^46 55 57^ In Kenyan settings, background levels of social support also rose among non-beneficiaries between 2018 and 2019, perhaps indicating some spill-over effects and that such support can increase as individuals age, and this diluted the effects observed compared with beneficiaries.

### Aspirations and expectations

It was encouraging that aspirations and, although to a lesser extent, expectations, were high. This suggests that intervention approaches should focus on helping AGYW to realise their goals, through strengthening of individual and collective agency and access to relevant resources. Given the high levels of aspiration, it was not surprising that there was little difference by DREAMS invitation. Nonetheless, differences by DREAMS invitation status for some expectations related to education, employment and health, as well as qualitative research conducted in the same/similar settings,^58 65 66^ support the potential of DREAMS, and other interventions, to make a positive contribution to change.

The heterogeneity observed by setting mirrored the different cultural contexts. For example, aspirations around marriage were seen as more important in Kenya compared with uMkhanyakude, where marriage is now uncommon in the Zulu population.^67^ A context-specific understanding of aspirations and how they shape social identities will be important for guiding both DREAMS and wider sexual and reproductive health programming.^68^

### Study strengths and limitations

Representative samples of AGYW drawn from established demographic platforms, high cohort retention and detailed data collection on exposure to DREAMS and social outcome measures that was harmonised across settings, were key strengths of this study. We also used a range of robust, analytical approaches to control for confounding, with consistency in findings.

Limitations included differential loss to follow-up by AGYW characteristics, potentially contributing to selection bias. High cohort retention suggests the extent of any bias would be small, and our estimates of the impact of DREAMS were controlled for confounding variables measured at enrolment. Nevertheless, it is possible that outcomes among one or both of DREAMS invitees and non-invitees were different among individuals who were not followed up compared with those who were, even after controlling for characteristics at baseline. Differential reporting bias is also possible, if DREAMS beneficiaries were aware of programme aims and more likely to report favourable responses to questions on support networks, self-efficacy beliefs and aspirations, although use of independent (not part of DREAMS implementation) interviewers and assurances of confidentiality should have limited this bias. Misclassification of exposure and outcome may have occurred due to reliance on self-reported data. For example, the proportion defined as beneficiaries may be underestimated if AGYW did not self-identify as DREAMS invitees. This is relatively unlikely in Kenyan settings where invitation to DREAMS was coordinated by a single implementing partner, but could plausibly have occurred in uMkhanyakude.

Composite measures of social support and self-efficacy were informed by detailed exploratory analyses, prior to conducting the impact analyses, and based on established scale items or questions relevant to programming, although choice of cut-offs may have influenced findings. Our outcome measures were intended to capture important aspects of individual and collective agency, but we did not assess others such as self-esteem, reflection, decision-making processes or the ability to negotiate or take on a leadership role. Nor did we assess the broader contextual factors—institutional and social structures, and access to resources beyond health services—that shape AGYW choices and actions, and are included in models of empowerment,^4 6 7 13^ although parallel analyses are being conducted on the impact of DREAMS on gender norms in our study population.^69^ Measurement of these constructs through structured questionnaires is challenging, for example, measuring ‘resources’ beyond simple access indicators,^4^ and further research is needed to develop and apply context-appropriate measures to more fully assess the impacts of DREAMS on AGYW empowerment. This includes further development of context-specific measures of self-efficacy, social support and aspirations. Further qualitative research to more thoroughly explore how DREAMS may have contributed to and influenced the process of empowerment, including how AGYW navigated challenges and societal structures, is also underway.

Our results may not be generalisable to all DREAMS districts, but represent diverse implementation contexts and can contribute important insights for other settings implementing DREAMS.

## Conclusion

We have identified encouraging impacts of the real-world implementation of the DREAMS package on aspects of AGYW empowerment, particularly social support and connectedness, in a range of contexts. Such outcomes are important in their own right to the well-being of young women in sub-Saharan Africa, and contribute to accelerating sustainable development goals.^70^ Weaker and more heterogeneous findings for self-efficacy and for impacts among older AGYW, highlight that opportunities remain to strengthen and sustain DREAMS programming to increase empowerment, particularly among young women.

## Data Availability

Data are available on reasonable request. Data underlying published results will be accessible and open, subject to a transition period (available from the London School of Hygiene and Tropical Medicine data repository https://datacompass.lshtm.ac.uk by contacting researchdatamanagement@lshtm.ac.uk), as per the Open Access Policy of the Bill & Melinda Gates Foundation.
