# Supplementary material for: Impact of the DREAMS Partnership on social support and general self-efficacy among adolescent girls and young women: causal analysis of population-based cohorts in Kenya and South Africa
Source: BMJ Glob Health. 2022 Mar 1;7(3):e006965. doi: 10.1136/bmjgh-2021-006965 (PMC8889325; doi:10.1136/bmjgh-2021-006965)
Supplement: Supplementary data [file bmjgh-2021-006965supp001.pdf]

Supplementary file 1. DREAMS Core Package

Level	Package level	Package category	Target group(s)	Description of intervention activities, with examples
Individual level	Empower AGYW and reduce their risk	**HIV Testing Services**	AGYW & male partners	HIV testing; linkage to care & ART if positive, or linkage to other DREAMS prevention if negative
		**Social asset building**	AGYW	Build social skills and networks; connect AGYW with peers & adults, for information, emotional & material support
		**Expand contraceptive mix**	AGYW	Promote use of modern contraception, dual methods alongside condoms, to reduce unplanned pregnancy and school drop-out
		**Condom promotion & provision**	AGYW & male partners	Increasing consistent use & availability, e.g. through condom distribution, adolescent-friendly SRH services
		**Post-violence care**	AGYW experienced/ at risk for violence	Youth-friendly screening & care for intimate partner violence/ violence against children, PEP
		**PrEP **selected countries***	AGYW at highest risk of acquiring HIV	Education on and targeted provision of PrEP (e.g. sex workers in South Africa), linkage to support services
Contextual level	Strengthen families	**Social protection**	AGYW & parents/guardians	Educational subsidies, combination socio-economic approaches e.g. savings groups
		**Parenting/caregiver programmes**	AGYW & parents / care-givers of AGYW	Parenting programmes on adolescent sexual/risk behaviours & protection from violence
	Mobilise community for change	**School-based HIV prevention**	AGYW & boys in schools	HIV & sex education, violence prevention education in schools
		**Community mobilisation & norms change**	AGYW, boys & men, broader communities	Community-based HIV and violence prevention programmes, social/gender norms change & gender-related messaging
	Reduce risk in male sex partners	**Characterisation of male sex partners to target interventions**	Sexual partners of AGYW	Target highly effective HIV prevention, care and treatment interventions. Develop services men are more likely to use. Research & characterise 'typical' partners of AGYW.
