# Supplementary material for: Impact of the DREAMS Partnership on social support and general self-efficacy among adolescent girls and young women: causal analysis of population-based cohorts in Kenya and South Africa
Source: BMJ Glob Health. 2022 Mar 1;7(3):e006965. doi: 10.1136/bmjgh-2021-006965 (PMC8889325; doi:10.1136/bmjgh-2021-006965)
Supplement: Supplementary data [file bmjgh-2021-006965supp002.pdf]

Supplementary file 2. Proportions with different aspects of social support* in 2019, by invitation to DREAMS and age group, among those followed-up in 2019

a(i).Gem

	Age 13-22		Age 13-17		Age 18-22	
	Never invited (N=436)	Invited in 2018 (N=582)	Never invited (N=261)	Invited in 2018 (N=361)	Never invited (N=175)	Invited in 2018 (N=221)
	% (col)	% (col)	% (col)	% (col)	% (col)	% (col)
**a) Is there a female in your community from whom you can borrow money in an emergency**						
Yes	32.1	38.0	27.6	34.1	38.9	44.3
No	67.9	62.0	72.4	65.9	61.1	55.7
Don't know	0.0	0.0	0.0	0.0	0.0	0.0
**b) Do you have a safe and private place to meet with girls and young women who are like you**						
Yes	34.2	55.3	33.7	54.3	34.9	57.0
No	64.9	44.3	65.1	45.2	64.6	43.0
Don't know	0.9	0.2	1.1	0.3	0.6	0.0
**d) Do you have at least one trusted female friend?**						
Yes	77.3	79.6	75.9	80.3	79.4	78.3
No	22.7	20.3	24.1	19.4	20.6	21.7
Don't know	0.0	0.0	0.0	0.0	0.0	0.0
**e) Do you know a woman in your community, other than a mother or guardian, whom you could turn to if you had a serious problem**						
Yes	50.2	52.4	46.7	50.4	55.4	55.7
No	49.8	47.3	53.3	49.3	44.6	43.9
Don't know	0.0	0.3	0.0	0.3	0.0	0.5

*Responses combined into composite social support binary outcome

Supplementary file 2. Proportions with different aspects of social support* in 2018, by invitation to DREAMS and age group, among those followed-up in 2018

a(ii).Gem

	Age 13-22		Age 13-17		Age 18-22	
	Never Invited (N=514)	Invited (N=657)	Never invited (N=285)	Invited (N=399)	Never invited (N=229)	Invited (N=258)
	% (col)	% (col)	% (col)	% (col)	% (col)	% (col)
**a) Is there a female in your community from whom you can borrow money in an emergency**						
Yes	28.0	32.1	25.6	28.1	31.0	38.4
No	72.0	67.6	74.4	71.7	69.0	61.2
Don't know	0.0	0.0	0.0	0.0	0.0	0.0
**b) Do you have a safe and private place to meet with girls and young women who are like you**						
Yes	28.0	65.0	28.1	64.4	27.9	65.9
No	68.7	34.1	67.7	34.8	69.9	32.9
Don't know	3.3	0.9	4.2	0.8	2.2	1.2
**d) Do you have at least one trusted female friend?**						
Yes	75.1	78.2	75.1	78.2	75.1	78.3
No	24.7	21.5	24.6	21.6	24.9	21.3
Don't know	0.2	0.3	0.4	0.3	0.0	0.4
**e) Do you know a woman in your community, other than a mother or guardian, whom you could turn to if you had a serious problem**						
Yes	47.7	49.3	44.9	46.1	51.1	54.3
No	51.8	50.1	54.4	53.4	48.5	45.0
Don't know	0.6	0.6	0.7	0.5	0.4	0.8

Supplementary file 2. Proportions with different aspects of social support* in 2019, by invitation to DREAMS and age group, among those followed-up in 2019

b(i).Nairobi

	Age 15-22		Age 15-17		Age 18-22	
	Never invited (N=224) % (col)	Invited by 2018 (N=628) % (col)	Never invited (N=95) % (col)	Invited by 2018 (N=369) % (col)	Never invited (N=129) % (col)	Invited by 2018 (N=259) % (col)
**a) Is there a female in your community from whom you can borrow money in an emergency?**						
Yes	50.4	56.8	47.4	57.2	52.7	56.4
No	49.1	43.2	51.6	42.8	47.3	43.6
Don't know	0.4	0.0	1.1	0.0	0.0	0.0
**b) Do you have a safe and private place to meet with girls and young women who are like you?**						
Yes	40.2	59.2	42.1	59.3	38.8	59.1
No	58.5	40.4	56.8	40.7	59.7	40.2
Don't know	1.3	0.3	1.1	0.0	1.6	0.8
**d) Do you have at least one trusted female friend?**						
Yes	76.8	82.6	75.8	87.0	77.5	76.4
No	22.8	17.2	23.2	13.0	22.5	23.2
Don't know	0.4	0.2	1.1	0.0	0.0	0.4
**e) Do you know a woman in your community, other than a mother or guardian, whom you could turn to if you had a serious problem?**						
Yes	62.1	65.8	56.8	66.1	65.9	65.3
No	37.9	34.2	43.2	33.9	34.1	34.7
Don't know	0.0	0.0	0.0	0.0	0.0	0.0

*Responses to each of the questions shown were combined into composite social support binary outcome

Supplementary file 2. Proportions with different aspects of social support* in 2018, by invitation to DREAMS and age group, among those followed-up in 2018

b(ii).Nairobi

	Age 15-22		Age 15-17		Age 18-22	
	Never invited (N=212)	Invited by 2018 (N=624)	Never invited (N=94)	Invited by 2018 (N=370)	Never invited (N=118)	Invited by 2018 (N=252)
	% (col)	% (col)	% (col)	% (col)	% (col)	% (col)
**a) Is there a female in your community from whom you can borrow money in an emergency?**						
Yes	51.9	57.8	51.1	57.8	52.6	57.8
No	47.6	41.7	47.9	41.4	47.4	42.2
Don't know	0.5	0.5	1.1	0.8	0.0	0.0
**b) Do you have a safe and private place to meet with girls and young women who are like you?**						
Yes	33.3	54.6	25.5	56.5	39.7	51.8
No	66.7	45.3	74.5	43.3	60.3	48.2
Don't know	0.0	0.2	0.0	0.3	0.0	0.0
**d) Do you have at least one trusted female friend?**						
Yes	72.4	77.9	77.7	80.9	68.1	73.5
No	27.6	22.1	22.3	19.1	31.9	26.5
Don't know	0.0	0.0	0.0	0.0	0.0	0.0
**e) Do you know a woman in your community, other than a mother or guardian, whom you could turn to if you had a serious problem?**						
Yes	52.9	61.0	46.8	60.2	57.8	62.3
No	47.1	39.0	53.2	39.8	42.2	37.8
Don't know	0.0	0.0	0.0	0.0	0.0	0.0

Supplementary file 2. Proportions with different aspects of social support* in 2019, by invitation to DREAMS and age group, among those followed-up in 2019

c(i).uMkhanyakude

	Age 13-22		Age 13-17		Age 18-22	
	Never invited (N=809)	Invited by 2018 (N=903)	Never invited (N=364)	Invited by 2018 (N=608)	Never invited (N=445)	Invited by 2018 (N=295)
	% (col)	% (col)	% (col)	% (col)	% (col)	% (col)
**a) Is there a female in your community from whom you can borrow money in an emergency?**						
Yes	56.5	54.6	48.6	50.3	62.9	63.4
No	43.4	45.3	51.4	49.5	36.9	36.6
Don't know/prefer not to answer	0.1	0.1	0.0	0.2	0.2	0.0
**b) Do you have a safe and private place to meet with girls and young women who are like you?**						
Yes	21.1	27.1	26.1	31.4	17.1	18.3
No	78.0	72.6	73.1	68.4	82.0	81.4
Don't know/prefer not to answer	0.9	0.2	0.8	0.2	0.9	0.3
**d) Do you have at least one trusted female friend?**						
Yes	78.1	82.5	83.0	87.3	74.2	72.5
No	21.8	17.2	17.0	12.5	25.6	26.8
Don't know/prefer not to answer	0.1	0.3	0.0	0.2	0.2	0.7
**e) Do you know a woman in your community, other than a mother or guardian, whom you could turn to if you had a serious problem?**						
Yes	69.2	68.5	69.2	68.4	69.2	68.8
No	30.7	30.9	30.5	31.1	30.8	30.5
Don't know/prefer not to answer	0.1	0.6	0.3	0.5	0.0	0.7

*Responses to each of the questions shown were combined into composite social support binary outcome

Supplementary file 2. Proportions with different aspects of social support* in 2018, by invitation to DREAMS and age group, among those followed-up in 2018

c(ii).uMkhanyakude

	Age 13-22		Age 13-17		Age 18-22	
	Never invited (N=886)	Invited by 2018 (N=966)	Never invited (N=389)	Invited by 2018 (N=651)	Never invited (N=497)	Invited by 2018 (N=315)
	% (col)	% (col)	% (col)	% (col)	% (col)	% (col)
**a) Is there a female in your community from whom you can borrow money in an emergency?**						
Yes	56.7	51.3	53.2	47.6	59.4	59.0
No	42.8	48.3	46.3	52.1	40.0	40.6
Don't know/prefer not to answer	0.6	0.3	0.5	0.3	0.6	0.3
**b) Do you have a safe and private place to meet with girls and young women who are like you?**						
Yes	14.1	34.8	17.7	41.3	11.3	21.3
No	85.7	65.1	82.3	58.5	88.3	78.7
Don't know/prefer not to answer	0.2	0.1	0.0	0.2	0.4	0.0
**d) Do you have at least one trusted female friend?**						
Yes	79.0	81.7	84.6	83.9	74.6	77.1
No	20.8	18.3	15.4	16.1	24.9	22.9
Don't know/prefer not to answer	0.2	0.0	0.0	0.0	0.4	0.0
**e) Do you know a woman in your community, other than a mother or guardian, whom you could turn to if you had a serious problem?**						
Yes	66.1	64.5	68.1	64.4	64.6	64.8
No	33.7	35.5	31.9	35.6	35.2	35.2
Don't know/prefer not to answer	0.1	0.0	0.0	0.0	0.2	0.0
