# Supplementary material for: Impact of the DREAMS Partnership on social support and general self-efficacy among adolescent girls and young women: causal analysis of population-based cohorts in Kenya and South Africa
Source: BMJ Glob Health. 2022 Mar 1;7(3):e006965. doi: 10.1136/bmjgh-2021-006965 (PMC8889325; doi:10.1136/bmjgh-2021-006965)
Supplement: Supplementary data [file bmjgh-2021-006965supp003.pdf]

Supplementary file 3. Distribution of responses to each statement from the general self efficacy scale in 2018, by age group and invitation to participate in DREAMS, among AGYW followed up in 2019.

a(i). Gem

	Overall	Age group at cohort enrolment		Invited to DREAMS in 2018	
	Total (N=1171)	13-17 (N=684)	18-22 (N=487)	Never invited (N=514)	Invited in 2018 (N=657)
	%	%	%	%	%
**I can always solve difficult problems if I try hard enough**					
Not at all true	14.2	17.3	9.9	12.5	15.5
Hardly true	12.5	14.2	10.1	13	12
Moderately true	22.5	21.8	23.4	24.9	20.5
Exactly sure	50.9	46.8	56.7	49.6	51.9
**If someone is against me (opposes me), I can find the means and ways to get what I want**					
Not at all true	14.8	17.1	11.5	15.8	14
Hardly true	13.7	15.5	11.1	13.4	13.9
Moderately true	22.7	22.4	23.2	25.7	20.4
Exactly sure	48.8	45	54.2	45.1	51.8
**It is easy for me to stick to my aims and accomplish my goals**					
Not at all true	7.7	9.8	4.7	7.8	7.6
Hardly true	8	9.9	5.3	8.8	7.5
Moderately true	17.9	18.7	16.8	18.3	17.7
Exactly sure	66.4	61.5	73.1	65.2	67.3
**I am confident that I could handle unexpected events well**					
Not at all true	13.2	16.8	8	13.4	12.9
Hardly true	11.4	12	10.7	11.5	11.4
Moderately true	25.2	25.1	25.3	26.7	24
Exactly sure	50.2	46.1	56.1	48.4	51.6
**Thanks to my resourcefulness, I know how to manage unexpected (unforeseen) situations**					
Not at all true	13.1	16.2	8.6	15.8	11
Hardly true	12.6	13.5	11.3	11.7	13.2
Moderately true	28.1	27	29.6	27.4	28.6
Exactly sure	46.3	43.3	50.5	45.1	47.2
**I can solve most problems if I make the necessary effort**					
Not at all true	10.4	14.9	4.1	9.1	11.4
Hardly true	10.2	11.5	8.2	11.5	9.1
Moderately true	21.7	22.4	20.7	22.8	20.9
Exactly sure	57.7	51.2	66.9	56.6	58.6
**I can remain calm when facing difficulties because I can rely on my own coping abilities**					
Not at all true	16.6	20.5	11.1	14.8	18
Hardly true	12.9	13.9	11.5	13.8	12.2
Moderately true	22.3	21.2	23.8	20.2	23.9
Exactly sure	48.2	44.4	53.6	51.2	46
**When I am faced with a problem, I can usually find several solutions**					
Not at all true	14.5	18.7	8.6	12.8	15.8
Hardly true	12	13.5	9.9	13.6	10.7
Moderately true	23.1	22.1	24.4	25.5	21.2
Exactly sure	50.5	45.8	57.1	48.1	52.4
**If I am in trouble, I can usually think of a solution**					
Not at all true	10.2	13.9	4.9	9.9	10.4
Hardly true	9.8	11.3	7.8	11.1	8.8
Moderately true	21.9	22.8	20.7	24.1	20.2
Exactly sure	58.1	52	66.5	54.9	60.6
**I can usually handle whatever comes my way**					
Not at all true	26.5	31.3	19.7	26.1	26.8
Hardly true	14.9	14.8	15.2	15.8	14.3
Moderately true	24.9	22.2	28.5	26.3	23.7
Exactly sure	33.7	31.7	36.6	31.9	35.2

Supplementary file 3. Distribution of responses to each statement from the general self efficacy scale in 2019, by age group and invitation to participate in DREAMS, among AGYW followed up in 2019.

a(ii). Gem

	Overall	Age group at cohort enrolment		Invited to DREAMS in 2018	
	Total (N=1018)	13-17 (N=622)	18-22 (N=396)	Never invited (N=436)	Invited in 2018 (N=582)
	%	%	%	%	%
**I can always solve difficult problems if I try hard enough**					
Not at all true	10.3	12.2	7.3	9.4	11.0
Hardly true	13.6	15.6	10.4	13.8	13.4
Moderately true	25.1	24.6	26.0	25.9	24.6
Exactly sure	51.0	47.6	56.3	50.9	51.0
**If someone is against me (opposes me), I can find the means and ways to get what I want**					
Not at all true	11.7	12.4	10.6	9.6	13.2
Hardly true	12.8	14.1	10.6	12.8	12.7
Moderately true	22.4	21.2	24.2	22.5	22.3
Exactly sure	53.1	52.3	54.5	55.0	51.7
**It is easy for me to stick to my aims and accomplish my goals**					
Not at all true	4.0	4.2	3.8	3.9	4.1
Hardly true	10.8	11.4	9.8	10.3	11.2
Moderately true	18.5	18.8	17.9	17.0	19.6
Exactly sure	66.7	65.6	68.4	68.8	65.1
**I am confident that I could handle unexpected events well**					
Not at all true	9.1	10.1	7.6	10.6	8.1
Hardly true	16.1	17.0	14.6	15.1	16.8
Moderately true	26.4	25.2	28.3	28.2	25.1
Exactly sure	48.3	47.6	49.5	46.1	50.0
**Thanks to my resourcefulness, I know how to manage unexpected (unforeseen) situations**					
Not at all true	9.9	12.1	6.6	11.9	8.4
Hardly true	18.3	20.3	15.2	18.3	18.2
Moderately true	27.7	25.2	31.6	27.8	27.7
Exactly sure	44.1	42.4	46.7	42.0	45.7
**I can solve most problems if I make the necessary effort**					
Not at all true	7.1	8.7	4.5	6.0	7.9
Hardly true	10.5	12.4	7.6	9.4	11.3
Moderately true	24.8	24.0	26.0	25.5	24.2
Exactly sure	57.7	55.0	61.9	59.2	56.5
**I can remain calm when facing difficulties because I can rely on my own coping abilities**					
Not at all true	16.1	19.1	11.4	19.3	13.7
Hardly true	16.6	18.6	13.4	15.1	17.7
Moderately true	24.3	22.3	27.3	22.9	25.3
Exactly sure	43.0	39.9	48.0	42.7	43.3
**When I am faced with a problem, I can usually find several solutions**					
Not at all true	9.6	11.1	7.3	10.6	8.9
Hardly true	11.6	12.4	10.4	12.2	11.2
Moderately true	27.3	27.8	26.5	26.8	27.7
Exactly sure	51.5	48.7	55.8	50.5	52.2
**If I am in trouble, I can usually think of a solution**					
Not at all true	5.3	6.8	3.0	5.3	5.3
Hardly true	9.4	10.5	7.8	7.6	10.8
Moderately true	24.6	24.9	24.0	23.9	25.1
Exactly sure	60.7	57.9	65.2	63.3	58.8
**I can usually handle whatever comes my way**					
Not at all true	24.4	27.2	19.9	25.5	23.5
Hardly true	17.6	19.1	15.2	17.7	17.5
Moderately true	26.6	23.2	32.1	26.1	27.0
Exactly sure	31.4	30.5	32.8	30.7	32.0

Supplementary file 3. Distribution of responses to each statement from the general self efficacy scale in 2018, by age group and invitation to participate in DREAMS, among AGYW followed up in 2019.

b(i). Nairobi

	Overall	Age group at cohort enrolment		Invited to DREAMS by 2018	
	Total (N=836)	15-17 (N=466)	18-22 (N=370)	Never invited (N=212)	Invited by 2018 (N=624)
	%	%	%	%	%
**I can always solve difficult problems if I try hard enough**					
Not sure	2	2.6	1.4	2.8	1.8
Not at all true	2.6	2.4	3.0	3.3	2.4
Hardly true	6.6	6.9	6.2	6.1	6.7
Moderately true	23.6	22.7	24.6	20.8	24.5
Exactly sure	64.6	65.5	63.5	66.0	64.1
Missing	0.6	0	1.4	0.9	0.5
**If someone is against me (opposes me), I can find the means and ways to get what I want**					
Not sure	3.2	3	3.5	5.2	2.6
Not at all true	8.9	7.7	10.3	14.2	7.1
Hardly true	11.7	12.9	10.3	10.4	12.2
Moderately true	20.5	21.5	19.2	20.8	20.4
Exactly sure	55.1	54.9	55.4	48.6	57.4
Missing	0.6	0	1.4	0.9	0.5
**It is easy for me to stick to my aims and accomplish my goals**					
Not sure	1.6	1.7	1.4	2.4	1.3
Not at all true	3.9	3.6	4.3	6.6	3.0
Hardly true	6.6	6	7.3	7.5	6.3
Moderately true	17.7	16.3	19.5	18.4	17.5
Exactly sure	69.6	72.3	66.2	64.2	71.5
Missing	0.6	0	1.4	0.9	0.5
**I am confident that I could handle unexpected events well**					
Not sure	4.2	4.5	3.8	5.2	3.8
Not at all true	5.1	4.7	5.7	7.5	4.3
Hardly true	11	10.9	11.1	10.8	11.1
Moderately true	28.8	28.1	29.7	29.2	28.7
Exactly sure	50.2	51.7	48.4	46.2	51.6
Missing	0.6	0	1.4	0.9	0.5
**Thanks to my resourcefulness, I know how to manage unexpected (unforeseen) situations**					
Not sure	3.5	3.9	3.0	4.2	3.2
Not at all true	5.5	4.9	6.2	7.1	5.0
Hardly true	11.2	12.2	10.0	9.9	11.7
Moderately true	28.1	27.5	28.9	26.9	28.5
Exactly sure	51.1	51.5	50.5	50.9	51.1
Missing	0.6	0	1.4	0.9	0.5
**I can solve most problems if I make the necessary effort**					
Not sure	1.3	1.9	0.5	2.4	1.0
Not at all true	2.5	1.7	3.5	3.8	2.1
Hardly true	7.5	6.9	8.4	6.1	8.0
Moderately true	19.7	19.7	19.7	21.2	19.2
Exactly sure	68.3	69.7	66.5	65.6	69.2
Missing	0.6	0	1.4	0.9	0.5
**I can remain calm when facing difficulties because I can rely on my own coping abilities**					
Not sure	2.3	2.6	1.9	3.3	1.9
Not at all true	5.1	4.7	5.7	7.1	4.5
Hardly true	8.0	8.6	7.3	8.5	7.9
Moderately true	23.6	25.5	21.1	21.2	24.4
Exactly sure	60.4	58.6	62.7	59	60.9
Missing	0.6	0	1.4	0.9	0.5
**When I am faced with a problem, I can usually find several solutions**					
Not sure	1.4	1.1	1.9	2.4	1.1
Not at all true	4.4	3.2	5.9	6.6	3.7
Hardly true	7.5	8.8	5.9	9.9	6.7
Moderately true	27	28.1	25.7	27.4	26.9
Exactly sure	59	58.8	59.2	52.8	61.1
Missing	0.6	0	1.4	0.9	0.5
**If I am in trouble, I can usually think of a solution**					
Not sure	1.2	1.3	1.1	1.9	1
Not at all true	3	2.6	3.5	3.8	2.7
Hardly true	5.4	6.4	4.1	5.2	5.4
Moderately true	23.2	24	22.2	24.5	22.8
Exactly sure	66.6	65.7	67.8	63.7	67.6
Missing	0.6	0	1.4	0.9	0.5
**I can usually handle whatever comes my way**					
Not sure	2.8	3	2.4	3.8	2.4
Not at all true	6.6	5.6	7.8	9	5.8
Hardly true	10	12	7.6	10.4	9.9
Moderately true	28.7	28.8	28.6	28.8	28.7
Exactly sure	51.3	50.6	52.2	47.2	52.7
Missing	0.6	0	1.4	0.9	0.5

Supplementary file 3. Distribution of responses to each statement from the general self efficacy scale in 2019, by age group and invitation to participate in DREAMS, among AGYW followed up in 2019.

b(ii). Nairobi

	Overall	Age group at cohort enrolment		Invited to DREAMS by 2018	
	Total (N=852)	15-17 (N=464)	18-22 (N=388)	Never invited (N=224)	Invited by 2018 (N=628)
	%	%	%	%	%
**I can always solve difficult problems if I try hard enough**					
Not sure	3.1	3.2	2.8	3.6	2.9
Not at all true	2.3	2.6	2.1	1.8	2.5
Hardly true	7.7	9.3	5.9	8.9	7.3
Moderately true	20.7	17.5	24.5	25.9	18.8
Exactly sure	66.2	67.5	64.7	59.8	68.5
**If someone is against me (opposes me), I can find the means and ways to get what I want**					
Not sure	2.5	2.4	2.6	2.7	2.4
Not at all true	7	7.5	6.4	8	6.7
Hardly true	12.7	11.2	14.4	15.2	11.8
Moderately true	22.3	22	22.7	23.2	22.0
Exactly sure	55.5	56.9	53.9	50.9	57.2
**It is easy for me to stick to my aims and accomplish my goals**					
Not sure	1.3	1.3	1.3	1.8	1.1
Not at all true	1.9	2.4	1.3	0.9	2.2
Hardly true	8.1	8.2	8	8.5	8.0
Moderately true	20.1	18.3	22.2	27.2	17.5
Exactly sure	68.7	69.8	67.3	61.6	71.2
**I am confident that I could handle unexpected events well**					
Not sure	5.2	5.6	4.6	4	5.6
Not at all true	3.9	5.2	2.3	3.1	4.1
Hardly true	9.4	8.8	10.1	9.8	9.2
Moderately true	25.5	26.3	24.5	32.1	23.1
Exactly sure	56.1	54.1	58.5	50.9	58.0
**Thanks to my resourcefulness, I know how to manage unexpected (unforeseen) situations**					
Not sure	3.6	3.4	3.9	3.1	3.8
Not at all true	4.5	5.2	3.6	2.2	5.3
Hardly true	12.1	12.3	11.9	16.5	10.5
Moderately true	25.5	24.1	27.1	29.5	24.0
Exactly sure	54.3	55	53.6	48.7	56.4
**I can solve most problems if I make the necessary effort**					
Not sure	0.8	0.6	1	0	1.1
Not at all true	2.3	2.2	2.6	1.8	2.5
Hardly true	7.7	9.3	5.9	8	7.6
Moderately true	19.8	17.9	22.2	23.2	18.6
Exactly sure	69.2	70	68.3	67.0	70.1
**I can remain calm when facing difficulties because I can rely on my own coping abilities**					
Not sure	3.6	3.7	3.6	4	3.5
Not at all true	5.2	6.5	3.6	3.6	5.7
Hardly true	8.8	9.3	8.2	7.1	9.4
Moderately true	22.7	22	23.5	22.8	22.6
Exactly sure	59.7	58.6	61.1	62.5	58.8
**When I am faced with a problem, I can usually find several solutions**					
Not sure	2.7	2.6	2.8	1.8	3.0
Not at all true	2.3	2.6	2.1	3.1	2.1
Hardly true	11.2	12.9	9	11.6	11.0
Moderately true	23.6	23.3	24	25.4	22.9
Exactly sure	60.2	58.6	62.1	58	61.0
**If I am in trouble, I can usually think of a solution**					
Not sure	2.3	2.4	2.3	2.2	2.4
Not at all true	1.9	2.4	1.3	2.7	1.6
Hardly true	7.7	8.8	6.4	9.8	7.0
Moderately true	21.1	20	22.4	25.9	19.4
Exactly sure	66.9	66.4	67.5	59.4	69.6
**I can usually handle whatever comes my way**					
Not sure	3.5	4.5	2.3	2.7	3.8
Not at all true	4.7	4.7	4.6	4	4.9
Hardly true	10.9	11.2	10.6	12.1	10.5
Moderately true	28.4	27.8	29.1	33.9	26.4
Exactly sure	52.5	51.7	53.4	47.3	54.3

Supplementary file 3. Distribution of responses to each statement from the general self efficacy scale in 2018, by age group and invitation to participate in DREAMS, among AGYW followed up in 2019.

c(i). uMkhanyakude

	Overall	Age group at cohort enrolment		Invited to DREAMS by 2018	
	Total (N=1853)	13-17 (N=1041)	18-22 (N=812)	Never invited (N=886)	Invited by 2018 (N=967)
	%	%	%	%	%
**I can always solve difficult problems if I try hard enough**					
Not at all true	22.5	25.3	18.8	22.9	22
Hardly true	14.1	14.9	13.1	13.5	14.6
Moderately true	11.6	12.7	10.2	13.2	10.1
Exactly sure	51.9	47.2	57.9	50.3	53.3
**If someone is against me (opposes me), I can find the means and ways to get what I want**					
Not at all true	20.5	21.9	18.6	21.4	19.5
Hardly true	15.3	17.6	12.3	14.9	15.6
Moderately true	11.3	12.2	10.1	11.9	10.8
Exactly sure	53	48.3	59	51.8	54.1
**It is easy for me to stick to my aims and accomplish my goals**					
Not at all true	14.4	14.5	14.2	15.3	13.4
Hardly true	11.8	12.6	10.7	13	10.7
Moderately true	10	10.8	9.1	11.5	8.7
Exactly sure	63.8	62.2	66	60.2	67.2
**I am confident that I could handle unexpected events well**					
Not at all true	17.2	17.8	16.5	17.9	16.5
Hardly true	11.8	13.4	9.6	11.4	12.1
Moderately true	11.5	13.1	9.6	13.7	9.6
Exactly sure	59.5	55.7	64.3	57	61.7
**Thanks to my resourcefulness, I know how to manage unexpected (unforeseen) situations**					
Not at all true	19.4	21.6	16.5	19.9	18.9
Hardly true	12.3	12.9	11.5	12	12.5
Moderately true	12.3	13.3	11	13.1	11.5
Exactly sure	56.1	52.3	61.1	55.1	57.1
**I can solve most problems if I make the necessary effort**					
Not at all true	26.6	29.7	22.5	27.7	25.5
Hardly true	12.4	12	12.9	11.5	13.2
Moderately true	10.7	11	10.5	12	9.6
Exactly sure	50.3	47.4	54.1	48.9	51.6
**I can remain calm when facing difficulties because I can rely on my own coping abilities**					
Not at all true	12.8	12.8	12.8	12.9	12.7
Hardly true	10.7	11.6	9.5	10.5	10.9
Moderately true	9.8	11.5	7.6	10.8	8.9
Exactly sure	66.7	64.1	70.1	65.8	67.5
**When I am faced with a problem, I can usually find several solutions**					
Not at all true	10.4	12.9	7.1	9.7	11
Hardly true	14.3	14.7	13.8	14.3	14.3
Moderately true	10.8	12.3	8.9	11.2	10.4
Exactly sure	64.5	60.1	70.2	64.8	64.3
**If I am in trouble, I can usually think of a solution**					
Not at all true	12.4	15.1	9	11.4	13.3
Hardly true	13.4	13	14	13.9	13
Moderately true	11.4	12.9	9.6	12.1	10.9
Exactly sure	62.7	59.1	67.4	62.6	62.8
**I can usually handle whatever comes my way**					
Not at all true	25.4	25.4	25.4	28.7	22.3
Hardly true	11.6	13.8	8.7	10.7	12.4
Moderately true	11.9	13.3	10.2	13.1	10.9
Exactly sure	51.1	47.6	55.7	47.5	54.4

Supplementary file 3. Distribution of responses to each statement from the general self efficacy scale in 2019, by age group and invitation to participate in DREAMS, among AGYW followed up in 2019.

c(ii). uMkhanyakude

	Overall	Age group at cohort enrolment		Invited to DREAMS by 2018	
	Total (N=1712)	13-17 (N=972)	18-22 (N=740)	Never invited (N=809)	Invited by 2018 (N=903)
	%	%	%	%	%
**I can always solve difficult problems if I try hard enough**					
Not at all true	19.9	24.4	13.9	20.4	19.4
Hardly true	8.7	9.6	7.6	9.3	8.2
Moderately true	17.2	17.6	16.6	16.4	17.8
Exactly sure	54.3	48.5	61.9	53.9	54.6
**If someone is against me (opposes me), I can find the means and ways to get what I want**					
Not at all true	17.5	18.5	16.2	18.4	16.7
Hardly true	11.2	12.8	9.1	10.9	11.4
Moderately true	15.2	15.7	14.6	16.2	14.4
Exactly sure	56.1	53	60.1	54.5	57.5
**It is easy for me to stick to my aims and accomplish my goals**					
Not at all true	10	10.1	10	10	10.1
Hardly true	9.9	11.4	8	10.1	9.7
Moderately true	13.4	13.4	13.5	15.7	11.4
Exactly sure	66.6	65.1	68.5	64.2	68.8
**I am confident that I could handle unexpected events well**					
Not at all true	14	16.4	10.9	13.8	14.2
Hardly true	10.6	12.4	8.1	10.6	10.5
Moderately true	16.5	17.3	15.5	17.8	15.4
Exactly sure	58.9	53.9	65.4	57.7	59.9
**Thanks to my resourcefulness, I know how to manage unexpected (unforeseen) situations**					
Not at all true	16.4	19.7	12	16.8	15.9
Hardly true	11.6	13	9.9	11.5	11.7
Moderately true	17	17.5	16.4	18	16.1
Exactly sure	55	49.9	61.8	53.6	56.3
**I can solve most problems if I make the necessary effort**					
Not at all true	18.8	22.7	13.6	18.9	18.7
Hardly true	13.3	13.8	12.6	14.1	12.5
Moderately true	15.1	15.3	14.7	15.2	15
Exactly sure	52.9	48.1	59.1	51.8	53.8
**I can remain calm when facing difficulties because I can rely on my own coping abilities**					
Not at all true	8.4	9.6	6.8	8.7	8.1
Hardly true	10.3	11.8	8.2	10.8	9.9
Moderately true	12.8	13.1	12.4	13.7	12
Exactly sure	68.6	65.5	72.6	66.9	70.1
**When I am faced with a problem, I can usually find several solutions**					
Not at all true	7	9.1	4.2	6.6	7.3
Hardly true	13.3	16	9.6	14.5	12.2
Moderately true	15.1	14.5	15.9	15.2	15.1
Exactly sure	64.7	60.4	70.3	63.8	65.4
**If I am in trouble, I can usually think of a solution**					
Not at all true	7.5	8.8	5.8	7.3	7.8
Hardly true	10.7	12.2	8.8	11.9	9.7
Moderately true	14.2	15.6	12.3	14.5	14
Exactly sure	67.5	63.3	73.1	66.4	68.5
**I can usually handle whatever comes my way**					
Not at all true	18.7	22.6	13.5	19.4	18.1
Hardly true	13.5	13.5	13.5	14.2	12.8
Moderately true	14.7	15.4	13.6	14.7	14.6
Exactly sure	53.2	48.5	59.3	51.7	54.5
