# Supplementary material for: Impact of the DREAMS Partnership on social support and general self-efficacy among adolescent girls and young women: causal analysis of population-based cohorts in Kenya and South Africa
Source: BMJ Glob Health. 2022 Mar 1;7(3):e006965. doi: 10.1136/bmjgh-2021-006965 (PMC8889325; doi:10.1136/bmjgh-2021-006965)

Supplementary file 4a. AGYW Cohort flow diagram: Gem

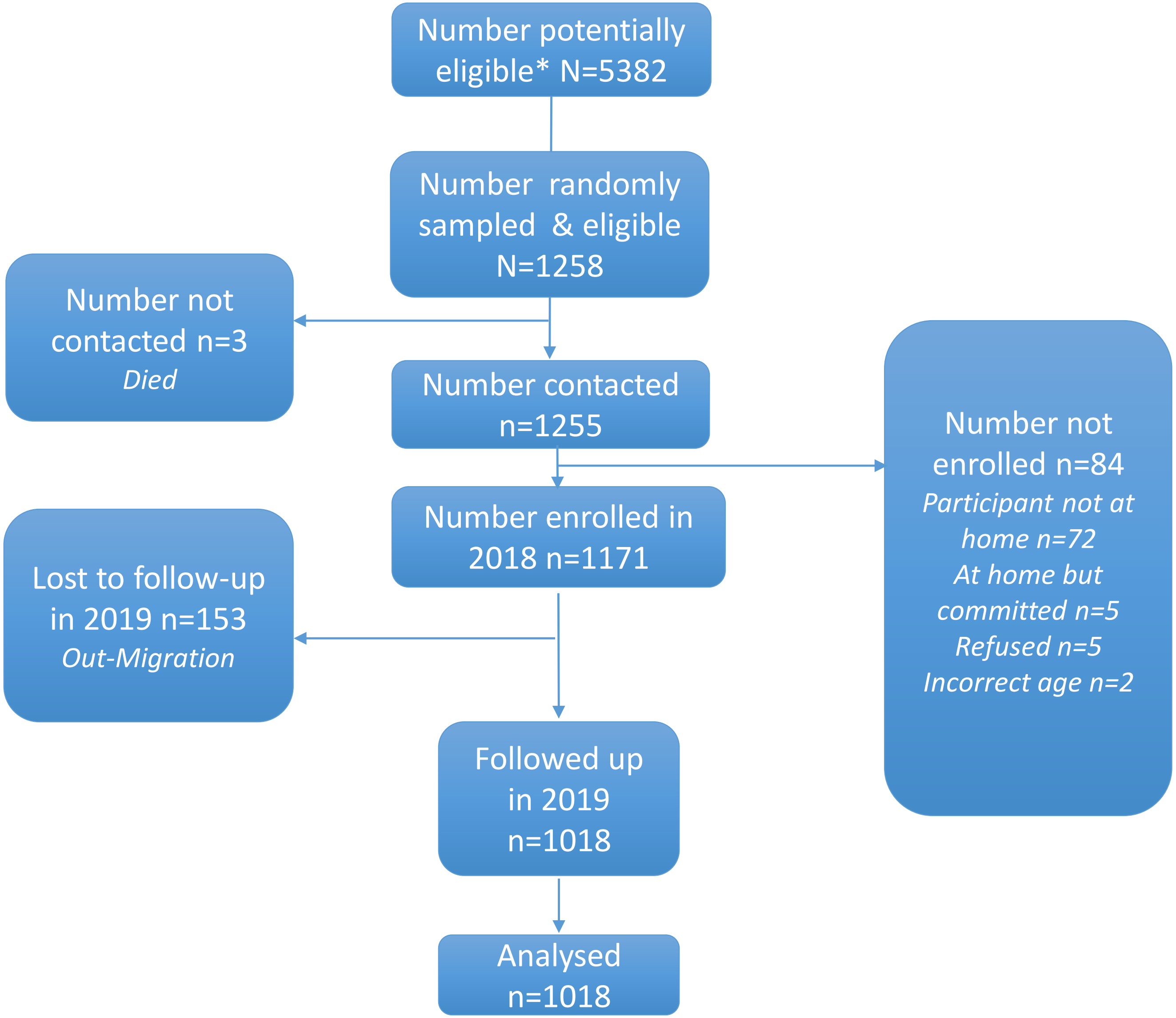

*All AGYW aged 13-22 resident in 2018 in households participating in population-based surveys in the Gem demographic surveillance system

Supplementary file 4b. AGYW cohort flow diagram: Nairobi

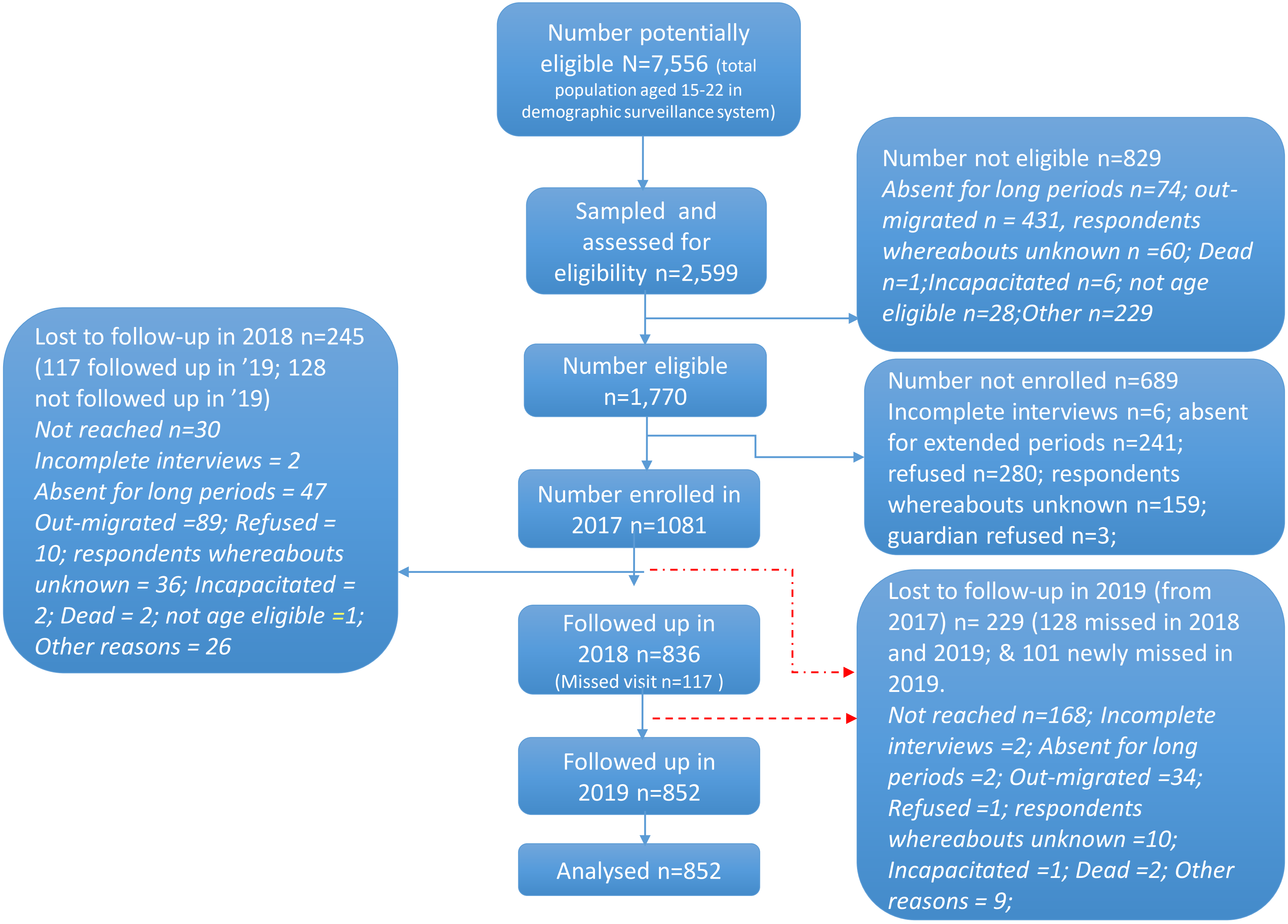

Supplementary file 4c. AGYW Cohort flow diagram: uMkhanyakude

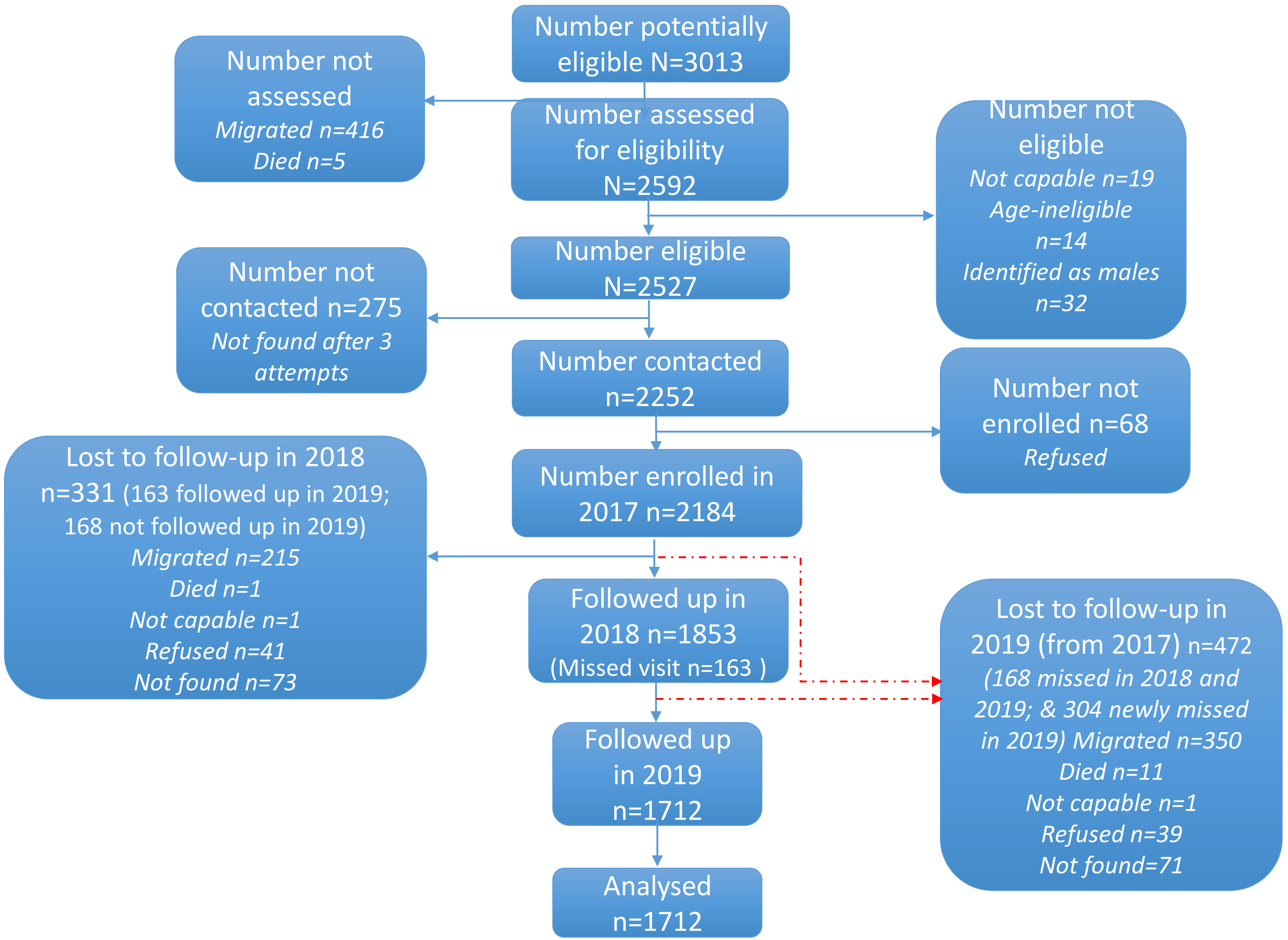

Supplement: Supplementary data [file bmjgh-2021-006965supp004.pdf]
