# Supplementary material for: Impact of the DREAMS Partnership on social support and general self-efficacy among adolescent girls and young women: causal analysis of population-based cohorts in Kenya and South Africa
Source: BMJ Glob Health. 2022 Mar 1;7(3):e006965. doi: 10.1136/bmjgh-2021-006965 (PMC8889325; doi:10.1136/bmjgh-2021-006965)
Supplement: Supplementary data [file bmjgh-2021-006965supp005.pdf]

Supplementary file 5: Proportions of AGYW retained in the study vs lost to follow up by 2019 (endline), by AGYW characteristics at study enrolment, in three settings

a. Gem

Characteristics at enrolment in 2018	2018	2019		
	Enrolled (N=1171) n (col%)	Followed up (N=1018) n (row %)	Lost to follow up (N=153) n (row %)	p value [a]
**Invited in 2018**				
No	514 (43.9)	436 (84.8)	78 (15.2)	0.058
Yes	657 (56.1)	582 (88.6)	75 (11.4)	
**Age group**				
13-17	684 (58.4)	622 (90.9)	62 (9.1)	<0.001
18-22	487 (41.6)	396 (81.3)	91 (18.7)	
**Educational attainment**				
Primary/None	481 (41.1)	435 (90.4)	46 (9.6)	<0.001
Secondary and above	411 (35.1)	372 (90.5)	39 (9.5)	
Unknown	279 (23.8)	211 (75.6)	68 (24.4)	
**Socio-economic status**				
Low	480 (41.0)	424 (88.3)	56 (11.7)	0.361
Middle	223 (19.0)	195 (87.4)	28 (12.6)	
High	468 (40.0)	399 (85.3)	69 (14.7)	
**Food insecurity***				
No	918 (78.4)	789 (85.9)	129 (14.1)	0.056
Yes	253 (21.6)	229 (90.5)	24 (9.5)	
**Ever had sex**				
No	766 (65.4)	701 (91.5)	65 (8.5)	<0.001
Yes	405 (34.6)	317 (78.3)	88 (21.7)	
**Ever been pregnant**				
No	956 (81.6)	859 (89.9)	97 (10.1)	<0.001
Yes	215 (18.4)	159 (74.0)	56 (26.0)	

a Chi square p-value comparing difference in proportions followed up between each category of characteristics at enrolment

* Food insecurity was defined based on this question; In the past 4 weeks, did you or any household member go to sleep at night hungry because there was not enough food? With responses(Yes, No)

Supplementary file 5: Proportions of AGYW retained in the study vs lost to follow up by 2019 (endline), by AGYW characteristics at study enrolment, in three settings

b. Nairobi

Characteristics at enrolment in 2017	2017	2019		
	Enrolled (N=1081)	Followed up (N=852)	Lost to follow up (N=229)	p-value [a]
	n (col %)	n (row %)	n (row %)	
**Invited in 2017**				
No	545 (50.4)	400 (73.4)	145 (26.6)	
Yes	536 (49.6)	452 (84.3)	84 (15.7)	<0.001
**Age group**				
15-17	547 (50.6)	464 (84.8)	83 (15.2)	
18-22	534 (49.4)	388 (72.7)	146 (27.3)	<0.001
**Currently in school**				
No	455 (42.1)	312 (68.6)	143 (31.4)	
Yes	626 (57.9)	540 (86.3)	86 (13.7)	<0.001
**Socio-economic status**				
Poor	361 (33.4)	303 (83.9)	58 (16.1)	
Medium	360 (33.3)	277 (76.9)	83 (23.1)	0.013
Wealthy	360 (33.3)	272 (75.6)	88 (24.4)	
**Food insecure***				
No	730 (67.5)	564 (77.3)	166 (22.7)	
Yes	351 (32.5)	288 (82.1)	63 (17.9)	0.071
**Ever had sex**				
No	644 (59.6)	557 (86.5)	87 (13.5)	
Yes	437 (40.4)	295 (67.5)	142 (32.5)	<0.001
**Ever pregnant**				
No	782 (72.3)	647 (82.7)	135 (17.3)	
Yes	299 (27.7)	205 (68.6)	94 (31.4)	<0.001
**Marital status**				
Never married	843 (78.0)	695 (82.4)	148 (17.6)	
Ever married/co-habiting	238 (22.0)	157 (66.0)	81 (34.0)	<0.001

a Chi square p-value comparing difference in proportions followed up between each category of characteristics at enrolment

* Food insecurity was defined as using the question: In the past 4 weeks, did you or any household member go to sleep at night hungry because there was not enough food? (yes, no)

Supplementary file 5: Proportions of AGYW retained in the study vs lost to follow up by 2019 (endline), by AGYW characteristics at study enrolment, in three settings

c. uMkhanyakude

Characteristics at enrolment in 2017	2017	2019		
	Enrolled (N=2184)	Followed up (N=1712)	Lost to follow-up (N=472)	p-value [a]
	n (col %)	n (row %)	n (row %)	
**Invited in 2017**				
Yes	639 (29.3)	528 (82.6)	111 (17.4)	
No	1545 (70.7)	1184 (76.6)	361 (23.4)	0.002
**Age group**				
13-14	460 (21.1)	414 (90.0)	46 (10.0)	
15-17	688 (31.5)	558 (81.1)	130 (18.9)	
18-19	475 (21.8)	348 (73.3)	127 (26.7)	
20-22	561 (25.7)	392 (69.9)	169 (30.1)	<0.001
**Currently in school**				
No	540 (24.7)	359 (66.5)	181 (33.5)	
Yes	1644 (75.3)	1353 (82.3)	291 (17.7)	<0.001
**Socio-economic status***				
Low	727 (35.1)	592 (81.4)	135 (18.6)	
Middle	747 (36.0)	576 (77.1)	171 (22.9)	
High	600 (28.9)	479 (79.8)	121 (20.2)	0.116
**Food insecurity****				
No	1497 (68.7)	1175 (78.5)	322 (21.5)	
Yes	682 (31.3)	532 (78.0)	150 (22.0)	0.799
**Ever had sex**				
No	1278 (59.8)	1063 (83.2)	215 (16.8)	
Yes	861 (40.3)	615 (71.4)	246 (28.6)	<0.001
**Ever pregnant**				
No	1576 (73.0)	1275 (80.9)	301 (19.1)	
Yes	583 (27.0)	420 (72.0)	163 (28.0)	<0.001
**Migrated*****				
No	1781 (81.6)	1432 (80.4)	349 (19.6)	
Yes	403 (18.5)	280 (69.5)	123 (30.5)	<0.001

a Chi square p-value comparing difference in proportions followed up between each category of characteristics at enrolment

*110 missing values

** Food insecurity was defined as any report of reducing the size of food potions or skipping meals by any member of a household because there was not enough money to buy food in the past 12 months

***Migration was defined as any movement within or outside surveillance area since age of 13
