# Supplementary material for: Impact of the DREAMS Partnership on social support and general self-efficacy among adolescent girls and young women: causal analysis of population-based cohorts in Kenya and South Africa
Source: BMJ Glob Health. 2022 Mar 1;7(3):e006965. doi: 10.1136/bmjgh-2021-006965 (PMC8889325; doi:10.1136/bmjgh-2021-006965)
Supplement: Supplementary data [file bmjgh-2021-006965supp006.pdf]

Supplementary file 6. Sociodemographic characteristics of DREAMS beneficiaries and non-beneficiaries at the time of cohort enrolment, by age-group, among those followed-up in 2019

a. Gem

Characteristics at enrolment in 2018	Age 13-22 Overall (N=1018) % (col)	Never invited (N=436) % (col)	Invited in 2018 (N=582) % (col)	Age 13-17 Overall (N=622) % (col)	Never invited (N=261) % (col)	Invited in 2018 (N=361) % (col)	Age 18-22 Overall (N=396) % (col)	Never invited (N=175) % (col)	Invited in 2018 (N=221) % (col)
**Age**									
13-17	61.1	59.9	62.0						
18-22	38.9	40.1	38.0						
**Education completed**									
None/primary	42.7	40.1	44.7	57.4	52.1	61.2	19.7	22.3	17.6
Secondary/tertiary	36.5	32.8	39.3	23.3	23.4	23.3	57.3	46.9	65.6
Unknown	20.7	27.1	16.0	19.3	24.5	15.5	23.0	30.9	16.7
**Socio-economic status**									
Low	41.7	36.0	45.9	42.0	38.7	44.3	41.2	32.0	48.4
Middle	19.2	19.0	19.2	19.5	19.9	19.1	18.7	17.7	19.5
High	39.2	45.0	34.9	38.6	41.4	36.6	40.2	50.3	32.1
**Food insecure**									
No	77.5	82.6	73.7	79.4	83.5	76.5	74.5	81.1	69.2
Yes	22.5	17.4	26.3	20.6	16.5	23.5	25.5	18.9	30.8
**Orphanhood**									
No	60.4	59.4	61.2	63.7	62.8	64.3	55.3	54.3	56.1
Maternal	3.4	3.4	3.4	2.7	3.1	2.5	4.5	4.0	5.0
Paternal	9.0	8.3	9.6	8.5	9.2	8.0	9.8	6.9	12.2
Total	3.2	4.4	2.4	2.3	3.1	1.7	4.8	6.3	3.6
Unknown	23.9	24.5	23.4	22.8	21.8	23.5	25.5	28.6	23.1
**Sexual and pregnancy history**									
Never had sex	68.9	64.0	72.5	88.6	84.7	91.4	37.9	33.1	41.6
Ever sex, never pregnant	15.5	17.4	14.1	9.0	11.9	6.9	25.8	25.7	25.8
Ever pregnant	15.6	18.6	13.4	2.4	3.4	1.7	36.4	41.1	32.6

Supplementary file 6. Sociodemographic characteristics of DREAMS beneficiaries and non-beneficiaries at the time of cohort enrolment, by age-group, among those followed-up in 2019

b. Nairobi

Characteristics at enrolment in 2017	Age 15-22 Overall (N=852) % (col)	Never invited (N=224) % (col)	Invited by 2018 (N=628) % (col)	Age 15-17 Overall (N=464) % (col)	Never invited (N=95) % (col)	Invited by 2018 (N=369) % (col)	Age 18-22 Overall (N=388) % (col)	Never invited (N=129) % (col)	Invited by 2018 (N=259) % (col)
**Age**									
15-17	54.5	42.4	58.8						
18-22	45.5	57.6	41.2						
**DSS study site**									
Korogocho	60.2	63.8	58.9	60.8	72.6	57.7	59.5	57.4	60.6
Viwandani	39.8	36.2	41.1	39.2	27.4	42.3	40.5	42.6	39.4
**Ethnicity**									
Somali	8.9	7.1	9.6	9.9	5.3	11.1	7.7	8.5	7.3
Kamba	17.5	17.9	17.4	17.9	18.9	17.6	17	17.1	17
Kikuyu	31.9	27.2	33.6	32.5	31.6	32.8	31.2	24	34.7
Kisii	3.9	4.9	3.5	4.3	1.1	5.1	3.4	7.8	1.2
Luhya	15.8	16.1	15.8	16.4	16.8	16.3	15.2	15.5	15.1
Luo	15.7	17.4	15.1	14	18.9	12.7	17.8	16.3	18.5
Other	6.2	9.4	5.1	5	7.4	4.3	7.7	10.9	6.2
**Religion**									
Catholic	28.6	29.5	28.3	27.2	32.6	25.7	30.4	27.1	32
Other Christian	54.7	53.6	55.1	56	51.6	57.2	53.1	55	52.1
Muslim	14.4	15.2	14.2	14.7	13.7	14.9	14.2	16.3	13.1
No religion /other	2.2	1.8	2.4	2.2	2.1	2.2	2.3	1.6	2.7
**Currently in school**									
No	36.6	48.7	32.3	14.4	21.1	12.7	63.1	69	60.2
Yes	63.4	51.3	67.7	85.6	78.9	87.3	36.9	31	39.8
**Education completed**									
None/incomplete primary	10.8	13.4	9.9	14.2	20	12.7	6.7	8.5	5.8
Complete primary	20	24.1	18.5	22	27.4	20.6	17.5	21.7	15.4
Some secondary	48.1	33.9	53.2	60.1	45.3	64	33.8	25.6	37.8
Complete secondary/tertiary	21.1	28.6	18.5	3.7	7.4	2.7	42	44.2	40.9
**Self assessed household poverty**									
Very poor	13.5	10.3	14.6	12.5	8.4	13.6	14.7	11.6	16.2
Moderately poor	78.9	80.4	78.3	79.3	83.2	78.3	78.4	78.3	78.4
Not poor	7.6	9.4	7	8.2	8.4	8.1	7	10.1	5.4
**Socio-economic status**									
Low	35.6	34.4	36	37.5	37.9	37.4	33.2	31.8	34
Medium	32.5	35.3	31.5	33.6	40	32	31.2	31.8	30.9
High	31.9	30.4	32.5	28.9	22.1	30.6	35.6	36.4	35.1
**Food insecure**									
No	66.2	74.1	63.4	65.1	73.7	62.9	67.5	74.4	64.1
Yes	33.8	25.9	36.6	34.9	26.3	37.1	32.5	25.6	35.9
**Gender of household head**									
Male	61.5	64.3	60.5	59.1	62.1	58.3	64.4	65.9	63.7
Female	38.5	35.7	39.5	40.9	37.9	41.7	35.6	34.1	36.3
**AGYW is the household head**									
No	96.6	95.1	97.1	98.7	97.9	98.9	94.1	93	94.6
Yes	3.4	4.9	2.9	1.3	2.1	1.1	5.9	7	5.4
**Orphanhood**									
Not an orphan	77.8	75.9	78.5	80.2	78.9	80.5	75	73.6	75.7
Single/double orphan	22.2	24.1	21.5	19.8	21.1	19.5	25	26.4	24.3
**Sexual and pregnancy history**									
Never had sex	65.4	55.8	68.8	89.2	86.3	90	36.9	33.3	38.6
Ever sex, never pregnant	10.6	11.6	10.2	5.4	6.3	5.1	16.8	15.5	17.4
Ever pregnant	24.1	32.6	21	5.4	7.4	4.9	46.4	51.2	44
**Ever given birth**									
No	77.9	71	80.4	95.7	95.8	95.7	56.7	52.7	58.7
Yes	22.1	29	19.6	4.3	4.2	4.3	43.3	47.3	41.3

Supplementary file 6. Sociodemographic characteristics of DREAMS beneficiaries and non-beneficiaries at the time of cohort enrolment, by age-group, among those followed-up in 2019

c. uMkhanyakude

Characteristics at enrolment in 2017	Age 13-22 Overall (N=1712) % (col)	Never invited (N=809) % (col)	Invited by 2018 (N=903) % (col)	Age 13-17 Overall (N=972) % (col)	Never invited (N=364) % (col)	Invited by 2018 (N=608) % (col)	Age 18-22 Overall (N=740) % (col)	Never invited (N=445) % (col)	Invited by 2018 (N=295) % (col)
**Age group**									
13-14	24.2	20.4	27.6	42.6	45.3	41.0			
15-17	32.6	24.6	39.8	57.4	54.7	59.0			
18-19	20.3	23.2	17.7				47.0	42.2	54.2
20-22	22.9	31.8	15.0				53.0	57.8	45.8
**Current school/education level**									
In school: primary or less	8.9	6.1	11.4	15.5	13.2	16.9	0.1	0.2	0.0
In school: secondary/tertiary	70.2	63.4	76.3	83.4	85.4	82.2	52.8	45.3	64.1
Not in school: none or complete primary	1.4	2.2	0.7	1.0	1.4	0.8	3.0	3.8	1.7
Not in school: incomplete secondary	7.1	9.8	4.8				15.8	17.1	13.9
Not in school: complete secondary/tertiary	12.4	18.6	6.9				28.3	33.6	20.3
**Area**									
Rural	64.5	59.2	69.2	64.2	56.7	68.7	64.9	61.3	70.3
Peri-urban/urban	35.5	40.8	30.8	35.8	43.3	31.3	35.1	38.7	29.7
**Migrated**									
No	83.6	80.1	86.8	93.4	92.9	93.8	70.8	69.7	72.5
Yes	16.4	19.9	13.2	6.6	7.1	6.3	29.2	30.3	27.5
**Socio-economic status**									
Low	34.6	30.5	38.2	33.7	26.6	38.0	35.7	33.7	38.6
Medium	33.6	34.2	33.1	33.3	35.4	32.1	34.1	33.3	35.3
High	28.0	30.2	26.0	30.3	35.2	27.5	24.9	26.1	23.1
Unknown	3.8	5.1	2.7	2.6	2.7	2.5	5.4	7.0	3.1
**Food insecure**									
No	68.8	65.4	71.9	78.2	79.9	77.2	56.6	53.6	61.0
Yes	31.2	34.6	28.1	21.8	20.1	22.8	43.4	46.4	39.0
**Sexual and pregnancy history**									
Never had sex	62.4	53.3	70.6	89.5	89.5	89.4	27	23.7	31.9
Ever sex, never pregnant	12.8	14.5	11.2	5.7	5.0	6.1	22.1	22.3	21.7
Ever pregnant	24.8	32.2	18.2	4.9	5.5	4.5	50.9	54	46.4
**Violence**									
No	65.8	66.3	65.3	63.5	62.1	64.3	68.8	69.7	67.5
Yes	34.2	33.8	34.7	36.5	37.9	35.7	31.2	30.3	32.5
