# Supplementary material for: Impact of the DREAMS Partnership on social support and general self-efficacy among adolescent girls and young women: causal analysis of population-based cohorts in Kenya and South Africa
Source: BMJ Glob Health. 2022 Mar 1;7(3):e006965. doi: 10.1136/bmjgh-2021-006965 (PMC8889325; doi:10.1136/bmjgh-2021-006965)
Supplement: Supplementary data [file bmjgh-2021-006965supp007.pdf]

Supplementary file 7. Distribution of aspirations and expectations scores in 2019, among AGYW followed up in 2019, by age group and invitation to DREAMS

a. Gem

Aspirations (*"How important are the following things to you?"*)	Overall		13-17 (N=622)				18-22 (N=396)			
	Total (N=1018)		Not invited (N=261)		Invited in 2018 (N=361)		Not invited (N=175)		Invited in 2018 (N=221)	
	n	%	n	%	n	%	n	%	n	%
**Finishing secondary school**										
Not important at all	23	2.3	5	1.9	6	1.7	8	4.6	4	1.8
Not very important	21	2.1	5	1.9	5	1.4	3	1.7	8	3.6
Somewhat important	31	3.0	7	2.7	10	2.8	8	4.6	6	2.7
Very important	943	92.6	244	93.5	340	94.2	156	89.1	203	91.9
**Going to college/university**										
Not important at all	24	2.4	7	2.7	6	1.7	8	4.6	3	1.4
Not very important	47	4.6	10	3.8	19	5.3	8	4.6	10	4.5
Somewhat important	55	5.4	12	4.6	15	4.2	17	9.7	11	5.0
Very important	892	87.6	232	88.9	321	88.9	142	81.1	197	89.1
**Owning own home**										
Not important at all	18	1.8	6	2.3	7	1.9	4	2.3	1	0.5
Not very important	97	9.5	24	9.2	34	9.4	14	8.0	25	11.3
Somewhat important	75	7.4	11	4.2	30	8.3	13	7.4	21	9.5
Very important	828	81.3	220	84.3	290	80.3	144	82.3	174	78.7
**Having good job/ stable income**										
Not important at all	8	0.8	4	1.5	3	0.8	1	0.6	0	0.0
Not very important	12	1.2	4	1.5	5	1.4	2	1.1	1	0.5
Somewhat important	21	2.1	9	3.4	6	1.7	3	1.7	3	1.4
Very important	977	96.0	244	93.5	347	96.1	169	96.6	217	98.2
**Having children**										
Not important at all	24	2.4	7	2.7	11	3.0	4	2.3	2	0.9
Not very important	125	12.3	45	17.2	49	13.6	10	5.7	21	9.5
Somewhat important	108	10.6	23	8.8	40	11.1	17	9.7	28	12.7
Very important	761	74.8	186	71.3	261	72.3	144	82.3	170	76.9
**Getting married/finding a partner**										
Not important at all	43	4.2	7	2.7	22	6.1	7	4.0	7	3.2
Not very important	183	18.0	53	20.3	69	19.1	23	13.1	38	17.2
Somewhat important	126	12.4	28	10.7	47	13.0	22	12.6	29	13.1
Very important	666	65.4	173	66.3	223	61.8	123	70.3	147	66.5

**Expectations *("What are the chances that you will..?)***										
**Finish primary school**										
High (or already achieved)	921	90.5	237	90.8	330	91.4	153	87.4	201	91.0
About 50/50	49	4.8	16	6.1	25	6.9	1	0.6	7	3.2
Low	23	2.3	3	1.1	4	1.1	12	6.9	4	1.8
Not applicable	25	2.5	5	1.9	2	0.6	9	5.1	9	4.1
**Finish secondary school**										
High (or already achieved)	704	69.2	177	67.8	260	72.0	104	59.4	163	73.8
About 50/50	177	17.4	59	22.6	84	23.3	11	6.3	23	10.4
Low	90	8.8	16	6.1	14	3.9	36	20.6	24	10.9
Not applicable	47	4.6	9	3.4	3	0.8	24	13.7	11	5.0
**Go to university**										
High (or already achieved)	437	42.9	124	47.5	195	54.0	56	32.0	62	28.1
About 50/50	388	38.1	106	40.6	133	36.8	43	24.6	106	48.0
Low	144	14.1	23	8.8	30	8.3	52	29.7	39	17.6
Not applicable	49	4.8	8	3.1	3	0.8	24	13.7	14	6.3
**Have job that pays well**										
High (or already achieved)	424	41.7	125	47.9	184	51.0	43	24.6	72	32.6
About 50/50	517	50.8	126	48.3	162	44.9	103	58.9	126	57.0
Low	77	7.6	10	3.8	15	4.2	29	16.6	23	10.4
**Be able to own your own home**										
High (or already achieved)	516	50.7	139	53.3	181	50.1	89	50.9	107	48.4
About 50/50	454	44.6	115	44.1	161	44.6	78	44.6	100	45.2
Low	48	4.7	7	2.7	19	5.3	8	4.6	14	6.3
**Stay in good health most of time**										
High (or already achieved)	519	51.0	137	52.5	193	53.5	81	46.3	108	48.9
About 50/50	475	46.7	118	45.2	162	44.9	90	51.4	105	47.5
Low	24	2.4	6	2.3	6	1.7	4	2.3	8	3.6

Supplementary file 7. Distribution of aspirations and expectations scores in 2019, among AGYW followed up in 2019, by age group and invitation to DREAMS

b. Nairobi

**Aspirations (*"How important are the following things to you?"*)**	**Overall (2019)**		**15-17 (N=464)**				**18-22 (N=388)**			
	**Total (N=852)**		**Never invited by 2018 (N=95)**		**Invited by 2018 (N=369)**		**Never invited by 2018 (N=129)**		**Invited by 2018 (N=259)**	
	**n**	**%**	**n**	**%**	**n**	**%**	**n**	**%**	**n**	**%**
**Finishing secondary school**										
Not important at all	25	2.9	3	3.2	3	0.8	7	5.4	12	4.6
Not very important	32	3.8	8	8.4	11	3.0	8	6.2	5	1.9
Somewhat important	38	4.5	2	2.1	8	2.2	7	5.4	21	8.1
Very important	757	88.8	82	86.3	347	94.0	107	82.9	221	85.3
**Going to college/university**										
Not important at all	31	3.6	2	2.1	6	1.6	9	7.0	14	5.4
Not very important	50	5.9	8	8.4	17	4.6	10	7.8	15	5.8
Somewhat important	96	11.3	8	8.4	42	11.4	16	12.4	30	11.6
Very important	675	79.2	77	81.1	304	82.4	94	72.9	200	77.2
**Owning your own home**										
Not important at all	4	0.5	0	0.0	2	0.5	0	0.0	2	0.8
Not very important	18	2.1	3	3.2	6	1.6	0	0.0	9	3.5
Somewhat important	49	5.8	3	3.2	20	5.4	14	10.9	12	4.6
Very important	781	91.7	89	93.7	341	92.4	115	89.1	236	91.1
**Having a good job/stable income**										
Not important at all	2	0.2					1	0.8	1	0.4
Not very important	8	0.9	1	1.1	5	1.4	1	0.8	1	0.4
Somewhat important	28	3.3	5	5.3	9	2.4	5	3.9	9	3.5
Very important	814	95.5	89	93.7	355	96.2	122	94.6	248	95.8
**Having children**										
Not important at all	4	0.5	1	1.1	1	0.3	1	0.8	1	0.4
Not very important	41	4.8	4	4.2	25	6.8	4	3.1	8	3.1
Somewhat important	134	15.7	22	23.2	68	18.4	15	11.6	29	11.2
Very important	673	79.0	68	71.6	275	74.5	109	84.5	221	85.3
**Getting married/finding a partner**										
Not important at all	17	2.0	3	3.2	6	1.6	1	0.8	7	2.7
Not very important	84	9.9	12	12.6	43	11.7	7	5.4	22	8.5
Somewhat important	175	20.5	23	24.2	84	22.8	18	14.0	50	19.3
Very important	576	67.6	57	60.0	236	64.0	103	79.8	180	69.5

**Expectations *("What are the chances that you will..?)***										
**Finish primary school?**										
High(or already achieved)	816	95.8	93	97.9	363	98.4	118	91.5	242	93.4
About 50/50	5	0.6	0	0.0	1	0.3	0	0.0	4	1.5
Low	31	3.6	2	2.1	5	1.4	11	8.5	13	5.0
**Finish secondary school?**										
High(or already achieved)	599	70.3	67	70.5	295	79.9	75	58.1	162	62.5
About 50/50	76	8.9	10	10.5	35	9.5	2	1.6	29	11.2
Low	177	20.8	18	18.9	39	10.6	52	40.3	68	26.3
**Go to university?**										
High(or already achieved)	277	32.5	34	35.8	150	40.7	28	21.7	65	25.1
About 50/50	302	35.4	40	42.1	157	42.5	34	26.4	71	27.4
Low	273	32.0	21	22.1	62	16.8	67	51.9	123	47.5
**Have a job that pays well?**										
High(or already achieved)	356	41.8	43	45.3	172	46.6	45	34.9	96	37.1
About 50/50	423	49.6	49	51.6	179	48.5	66	51.2	129	49.8
Low	73	8.6	3	3.2	18	4.9	18	14.0	34	13.1
**Be able to own your own home?**										
High(or already achieved)	462	54.2	61	64.2	207	56.1	62	48.1	132	51.0
About 50/50	331	38.8	30	31.6	146	39.6	51	39.5	104	40.2
Low	59	6.9	4	4.2	16	4.3	16	12.4	23	8.9
**Stay in good health most of the time?**										
High(or already achieved)	535	62.8	57	60.0	225	61.0	80	62.0	173	66.8
About 50/50	301	35.3	38	40.0	137	37.1	47	36.4	79	30.5
Low	16	1.9	0	0.0	7	1.9	2	1.6	7	2.7

Supplementary file 7. Distribution of aspirations and expectations scores in 2019, among AGYW followed up in 2019, by age group and invitation to participate in DREAMS

c. uMkhanyakude

Aspirations (*"How important are the following things to you?"*)	Overall		13-17 (N=972)				18-22 (N=740)			
	Total (N=1712)		Never invited (N=364)		Invited by 2018 (N=608)		Never invited (N=445)		Invited by 2018 (N=295)	
	n	%	n	%	n	%	n	%	n	%
**Finishing secondary school**										
Not important at all	0	0.0	0	0.0	0	0.0	0	0.0	0	0.0
Not very important	5	0.3	1	0.3	0	0.0	3	0.7	1	0.3
Somewhat important	4	0.2	0	0.0	3	0.5	0	0.0	1	0.3
Important	409	23.9	81	22.3	132	21.7	115	25.9	81	27.5
Very important	1128	65.9	272	74.7	458	75.3	237	53.4	161	54.6
Already achieved	164	9.6	10	2.8	15	2.5	88	19.8	51	17.3
Don't know	1	0.1	0	0.0	0	0.0	1	0.2	0	0.0
**Going to college/university**										
Not important at all	3	0.2	1	0.3	0	0.0	2	0.5	0	0.0
Not very important	20	1.2	3	0.8	5	0.8	10	2.3	2	0.7
Somewhat important	13	0.8	0	0.0	6	1.0	4	0.9	3	1.0
Important	457	26.7	90	24.7	141	23.2	138	31.0	88	29.8
Very important	1202	70.2	270	74.2	453	74.5	282	63.4	197	66.8
Already achieved	12	0.7	0	0.0	2	0.3	6	1.4	4	1.4
Don't know	5	0.3	0	0.0	1	0.2	3	0.7	1	0.3
**Owning your own home**										
Not important at all	17	1.0	2	0.6	9	1.5	5	1.1	1	0.3
Not very important	75	4.4	20	5.5	31	5.1	16	3.6	8	2.7
Somewhat important	25	1.5	6	1.7	11	1.8	4	0.9	4	1.4
Important	646	37.7	119	32.7	219	36.0	177	39.8	131	44.4
Very important	942	55.0	217	59.6	337	55.4	239	53.7	149	50.5
Already achieved	1	0.1	0	0.0	0	0.0	0	0.0	1	0.3
Don't know	6	0.4	0	0.0	1	0.2	4	0.9	1	0.3
**Having a good job/stable income**										
Not important at all	1	0.1	0	0.0	0	0.0	0	0.0	1	0.3
Not very important	4	0.2	2	0.6	1	0.2	1	0.2	0	0.0
Somewhat important	14	0.8	3	0.8	5	0.8	6	1.4	0	0.0
Important	515	30.1	96	26.4	164	27.0	157	35.3	98	33.2
Very important	1176	68.7	263	72.3	438	72.0	279	62.7	196	66.4
Already achieved	1	0.1	0	0.0	0	0.0	1	0.2	0	0.0
Don't know	1	0.1	0	0.0	0	0.0	1	0.2	0	0.0
**Having children**										
Not important at all	276	16.1	87	24.0	98	16.1	51	11.5	40	13.6
Not very important	435	25.4	85	23.4	174	28.6	107	24.0	69	23.4
Somewhat important	103	6.0	25	6.9	41	6.7	27	6.1	10	3.4
Important	532	31.1	99	27.3	172	28.3	155	34.8	106	35.9
Very important	312	18.2	55	15.2	104	17.1	87	19.6	66	22.4
Already achieved	5	0.3	1	0.3	1	0.2	2	0.5	1	0.3
Don't know	48	2.8	11	3.0	18	3.0	16	3.6	3	1.0
**Getting married/finding a partner**										
Not important at all	256	15.0	72	19.8	84	13.8	58	13.1	42	14.2
Not very important	428	25.0	78	21.4	171	28.1	109	24.6	70	23.7

Somewhat important	96	5.6	26	7.1	38	6.3	21	4.7	11	3.7
Important	533	31.2	107	29.4	185	30.4	144	32.4	97	32.9
Very important	351	20.5	69	19.0	115	18.9	98	22.1	69	23.4
Already achieved	1	0.1	0	0.0	0	0.0	1	0.2	0	0.0
Don't know	46	2.7	12	3.3	15	2.5	13	2.9	6	2.0

**Expectations *("What are the chances that you will..?)***										
**Finish primary school?**										
High (or already achieved)	1693	98.9	362	99.5	604	99.3	435	97.8	292	99.0
About 50/50	5	0.3	0	0.0	3	0.5	2	0.5	0	0.0
Low	7	0.4	2	0.6	0	0.0	5	1.1	0	0.0
Don't know	7	0.4	0	0.0	1	0.2	3	0.7	3	1.0
**Finish secondary school?**										
High (or already achieved)	1498	87.5	325	89.3	537	88.3	378	84.9	258	87.5
About 50/50	133	7.8	32	8.8	58	9.5	25	5.6	18	6.1
Low	59	3.5	5	1.4	5	0.8	34	7.6	15	5.1
Don't know	22	1.3	2	0.6	8	1.3	8	1.8	4	1.4
**Go to university?**										
High (or already achieved)	984	57.5	258	70.9	371	61.0	205	46.1	150	50.9
About 50/50	395	23.1	72	19.8	154	25.3	101	22.7	68	23.1
Low	215	12.6	25	6.9	40	6.6	95	21.4	55	18.6
Don't know	118	6.9	9	2.5	43	7.1	44	9.9	22	7.5
**Have a job that pays well?**										
High (or already achieved)	957	55.9	240	65.9	368	60.5	201	45.2	148	50.2
About 50/50	460	26.9	88	24.2	164	27.0	131	29.4	77	26.1
Low	129	7.5	9	2.5	23	3.8	56	12.6	41	13.9
Don't know	166	9.7	27	7.4	53	8.7	57	12.8	29	9.8
**Be able to own your own home?**										
High (or already achieved)	995	58.1	234	64.3	385	63.3	216	48.5	160	54.2
About 50/50	454	26.5	94	25.8	143	23.5	135	30.3	82	27.8
Low	87	5.1	7	1.9	25	4.1	36	8.1	19	6.4
Don't know	176	10.3	29	8.0	55	9.1	58	13.0	34	11.5
**Stay in good health most of the time?**										
High (or already achieved)	898	52.5	205	56.3	347	57.1	185	41.6	161	54.6
About 50/50	491	28.7	105	28.9	170	28.0	140	31.5	76	25.8
Low	70	4.1	13	3.6	19	3.1	27	6.1	11	3.7
Don't know	253	14.8	41	11.3	72	11.8	93	20.9	47	15.9
