# Supplementary material for: Impact of the DREAMS Partnership on social support and general self-efficacy among adolescent girls and young women: causal analysis of population-based cohorts in Kenya and South Africa
Source: BMJ Glob Health. 2022 Mar 1;7(3):e006965. doi: 10.1136/bmjgh-2021-006965 (PMC8889325; doi:10.1136/bmjgh-2021-006965)
Supplement: Supplementary data [file bmjgh-2021-006965supp008.pdf]

Supplementary file 8. Conventional univariable and multivariable logistic regresssion model for the association between invited to DREAMS by 2018* with social support in 2019, showing all covariates.**

a. Gem

Variables at enrolment in 2018	Total N (1018)	Social support n (411)	%	Unadjusted OR OR	(95% CI)		p (LRT)	Fully adjusted OR*** aOR	(95% CI)		p (LRT)
**Invited to DREAMS**											
No	436	156	35.8	ref			0.01	ref			0.02
Yes	582	255	43.8	1.4	1.1	1.8		1.4	1.1	1.8	
**Age group**											
13-17	622	229	36.8	ref			0.004	ref			0.2
18-22	396	182	46	1.5	1.1	1.9		0.8	0.6	1.1	
**Educational attainment**											
Primary/None	435	146	33.6	ref				ref			<0.001
Secondary and above	372	181	48.7	1.9	1.4	2.5	<0.001	2	1.5	2.8	
Unknown	211	84	39.8	1.3	0.9	1.8		1.3	0.9	1.9	
**Socio-economic status**											
Low	424	197	46.5	ref				ref			0.2
Middle	195	71	36.4	0.7	0.5	0.9	0.004	0.7	0.5	1.1	
High	399	143	35.8	0.6	0.5	0.9		0.7	0.5	1.0	
**Orphanhood**											
No	615	246	40.0	ref				ref			0.9
Maternal	35	16	45.7	1.3	0.6	2.5		1.2	0.6	2.5	
Paternal	92	38	41.3	1.1	0.7	1.7	0.949	0.9	0.5	1.4	
Total	33	12	36.4	0.9	0.4	1.8		0.8	0.4	1.7	
Unknown	243	99	40.7	1.0	0.8	1.4		1.0	0.7	1.4	
**Food insecurity** [a]											
No	789	296	37.5	ref				ref			0.06
Yes	229	115	50.2	1.7	1.3	2.3	0.001	1.4	1.0	2.1	
**Sexual/pregnancy history**											
Never had sex	701	251	35.8	ref				ref			
Ever sex,never pregnant	158	77	48.7	1.7	1.2	2.4		1.7	1.1	2.4	<0.001
Ever pregnant	159	83	52.2	2.0	1.4	2.8	<0.001	2.1	1.4	3.3	

CI confidence interval; OR odds ratio; LRT likelihood ratio test

* Invited to DREAMS by 2018 defined as self-reported invitation to participate in DREAMS in 2018 (cohort enrolled in Gem in 2018) .

** Social support defined as answering 'yes' to at least 3 of the following 4 questions: "Is there a female in your community from whom you can borrow money in an emergency?; "Do you have at least one trusted female friend?"; "Do you know a woman in your community, other than a mother or guardian, whom you could turn to if you had a serious problem?"; "Do you have a safe and private place to meet with girls and young women who are like you?"

*** Fully adjusted model included: age, education, socio-economic status, orphanhood, food insecurity, sexual and pregnancy history

a Food insecurity was defined based on the question: In the past 4 weeks, did you or any household member go to sleep at night hungry because there was not enough food? With responses(Yes, No)

Supplementary file 8. Conventional univariable and multivariable logistic regresssion model for the association between invited to DREAMS by 2018* with social support in 2019, showing all covariates.**

b. Nairobi

Variables at enrolment in 2017	Total N (852)	Social support n (480)	row %	Unadjusted OR OR	(95% CI)		p (LRT)	Fully adjusted OR*** aOR	(95% CI)		p (LRT)
**Invited by 2018**											
No	224	111	49.6	ref				ref			
Yes	628	369	58.8	1.5	1.1	2.0	0.02	1.4	1.0	1.9	0.04
**Age group**											
15-17	464	266	57.3	ref							
18-22	388	214	55.2	0.9	0.7	1.2	0.5				
**Currently in school**											
No	312	166	53.2	ref							
Yes	540	314	58.2	1.2	0.9	1.6	0.2				
**Age-education**											
15-17 & in school: secondary/tertiary	272	174	64	ref				ref			
15-17 & in school: primary or less	125	62	49.6	0.6	0.4	0.9	0.001	0.7	0.4	1.0	0.06
15-17 & not in school	67	30	44.8	0.5	0.3	0.8		0.7	0.4	1.2	
18-22 & in school: tertiary or less	143	78	54.5	0.7	0.4	1.0		0.8	0.5	1.3	
18-22 & not in school: primary or less	89	39	43.8	0.4	0.3	0.7		0.6	0.3	1.3	
18-22 & not in school: incomplete secondary	55	31	56.4	0.7	0.4	1.3		1.2	0.5	2.5	
18-22 & not in school: complete secondary/tertiary	101	66	65.3	1.1	0.7	1.7		1.4	0.8	2.5	
**DSS study site**											
Korogocho	513	259	50.5	ref				ref			
Viwandani	339	221	65.2	1.8	1.4	2.4	<0.001	1.2	0.9	1.8	0.2
**Marital status**											
never married	695	392	56.4	ref				ref			
prev married/living w partner	24	11	45.8	0.7	0.3	1.5	0.5	1.3	0.5	3.5	0.2
curr married/living w partner	133	77	57.9	1.1	0.7	1.5		1.9	1.0	3.6	
**Sexual & pregnancy history**											
Never had sex	555	328	59.1	ref				ref			
Ever sex, never preg	90	45	50	0.7	0.4	1.1	0.07	0.6	0.4	1.1	0.03
Ever pregnant	205	105	51.2	0.7	0.5	1.0		0.5	0.2	0.9	
**Food insecure** [a]											
No	564	327	58	ref				ref			
Yes	288	153	53.1	0.8	0.6	1.1	0.2	1.1	0.8	1.5	0.6
**Socio-economic status**											
Poor	303	138	45.5	ref				ref			
Medium	277	160	57.8	1.6	1.2	2.3	<0.001	1.5	1.0	2.1	0.01
Wealthy	272	182	66.9	2.4	1.7	3.4		1.9	1.2	3.0	
**Self-assessed household poverty** [b]											
Very poor	115	63	54.8	ref				ref			
Moderately poor	672	380	56.5	1.1	0.7	1.6	0.9	1.0	0.7	1.5	1.0
Not poor	65	37	56.9	1.1	0.6	2.0		1.0	0.5	1.9	
**Orphanhood**											
No	663	378	57.0	ref				ref			
Yes (mother/both parents died)	189	102	54.0	0.9	0.6	1.2	0.6	1.0	0.7	1.4	1.0

CI confidence interval; OR odds ratio; LRT likelihood ratio test

* Invited to DREAMS by 2018 defined as self-reported invitation to participate in DREAMS in 2017 and/or 2018.

** Social support defined as answering 'yes' to at least 3 of the following 4 questions: "Is there a female in your community from whom you can borrow money in an emergency?; "Do you have at least one trusted female friend?"; "Do you know a woman in your community, other than a mother or guardian, whom you could turn to if you had a serious problem?"; "Do you have a safe and private place to meet with girls and young women who are like you?"

*** Fully adjusted model included: composite age-education variable; DSS study site; marital status; sexual and pregnancy history; socio-economic status; food insecurity; self-assessed household poverty; orphanhood

a Food insecurity was defined as using the question: In the past 4 weeks, did you or any household member go to sleep at night hungry because there was not enough food? (yes, no)

b Self-assessed household poverty was based on the responses to the question: In your opinion, how do you assess the economic situation of your household now? (Very poor, moderately poor or not poor)

Supplementary file 8. Conventional univariable and multivariable logistic regresssion model for the association between invited to DREAMS by 2018* with social support in 2019, showing all covariates.**

c. uMkhanyakude

Variables at enrolment in 2017	Total N (1712)	Social support n (778)	row %	Unadjusted OR OR	(95% CI)		p (LRT)	Fully adjusted OR*** aOR	(95% CI)		p (LRT)
**Invited by 2018**											
No	809	358	44.3	ref				ref			
Yes	903	420	46.5	1.1	0.9	1.3	0.3	1.1	0.9	1.3	0.5
**Age group**											
13-14	414	197	47.6	ref				ref			
15-17	558	244	43.7	0.9	0.7	1.1	0.7	0.9	0.7	1.1	0.5
18-19	348	159	45.7	0.9	0.7	1.2		1.0	0.7	1.5	
20-22	392	178	45.4	0.9	0.7	1.2		1.1	0.7	1.6	
**Area**											
Rural	1,095	518	47.3	ref				ref			
Peri-urban/urban	603	255	42.3	0.8	0.7	1.0	0.05	0.8	0.7	1.0	0.09
**Age 13-17, or 18-22 in school** [a]	1385	637	46.0	ref							
Age 13-17	972	441	45.4								
Age 18-22 and in school	391	185	47.3								
**incomplete secondary education** [b]								ref			
Yes	117	51	43.6	0.9	0.6	1.3	0.7	0.8	0.5	1.3	0.5
**completed secondary education** [c]								ref			
Yes	209	90	43.1	0.9	0.7	1.2	0.4	0.8	0.6	1.2	0.2
**Socio-economic status**											
Low	592	262	44.3	ref				ref			
Middle	576	281	48.8	1.2	1.0	1.5	0.3	1.2	1.0	1.5	0.3
High	479	207	43.2	1.0	0.8	1.2		1.0	0.7	1.2	
Unknown	65	28	43.1	1.0	0.6	1.6		1.0	0.6	1.7	
**Food insecure** [d]											
No	1,175	553	47.1	ref				ref			
Yes	532	223	41.9	0.8	0.7	1.0	0.05	0.8	0.6	1.0	0.04
**Migrated** [e]											
No	1,432	644	45	ref				ref			
Yes	280	134	47.9	1.1	0.9	1.5	0.4	1.2	0.9	1.6	0.2
**Sexual & pregnancy history**											
Never had sex	1,065	493	46.3	ref				ref			
Ever sex, never pregnant	218	86	39.4	0.8	0.6	1.0	0.1	0.8	0.6	1.1	0.2
Ever pregnant	423	199	47	1.0	0.8	1.3		1.1	0.8	1.4	

CI confidence interval; OR odds ratio; LRT likelihood ratio test

* Invited to DREAMS by 2018 defined as self-reported invitation to participate in DREAMS in 2017 and/or 2018.

** Social support defined as answering 'yes' to at least 3 of the following 4 questions: "Is there a female in your community from whom you can borrow money in an emergency?; "Do you have at least one trusted female friend?"; "Do you know a woman in your community, other than a mother or guardian, whom you could turn to if you had a serious problem?"; "Do you have a safe and private place to meet with girls and young women who are like you?"

*** Fully adjusted model included: age group in 4 categories, area, education (dummy variables 18-22 not in school and in/complete secondary - defined in 'b' and 'c' below), socio-economic status, food insecurity, migration, sexual & pregnancy history.

a Reference category for schooling dummy variables includes everyone who is in school, or 13-17 and out of school (very few, as almost all 13-17s are in school), or 18-22 and out of school with no/primary only education (few)

b Dummy variable, where 'no' (0) = all non-missing values of age and schooling, and those not coded as yes (not "18-22 not in school and incomplete secondary")

c Dummy variable, where 'no' (0) = all non-missing values of age and schooling, and those not coded as yes (not "18-22 not in school and complete secondary")

d Food insecurity was defined as any report of reducing the size of food potions or skipping meals by any member of a household because there was not enough money to buy food in the past 12 months

e Migration was defined as any movement within or outside surveillance area since age of 13

Supplementary file 8. Conventional univariable and multivariable logistic regresssion model for the association between invited to DREAMS by 2018* with self-efficacy in 2019, showing all covariates.**

d. Gem

Variables at enrolment in 2018	Total N (1018)	Self-efficacy n (351)	%	Unadjusted OR OR	(95% CI)		p (LRT)	Fully adjusted OR*** aOR	(95% CI)		p (LRT)
**Invited to DREAMS**											
No	436	145	33.3	Ref				Ref			
Yes	582	206	35.4	1.1	0.9	1.4	0.5	1.2	0.9	1.6	0.2
**Age group**											
13-17	622	187	30.1	Ref				Ref			
18-22	396	164	41.4	1.6	1.3	2.1	<0.001	1.0	0.7	1.4	0.8
**Educational attainment**											
Primary/None	435	103	23.7	Ref				Ref			
Secondary and above	372	171	46	2.7	2.0	3.7	<0.001	2.6	1.8	3.6	<0.001
Unknown	211	77	36.5	1.9	1.3	2.7		1.8	1.2	2.7	
**Socio-economic status**											
Low	424	136	32.1	Ref				Ref			
Middle	195	60	30.8	0.9	0.7	1.4	0.06	1.0	0.6	1.4	0.06
High	399	155	38.8	1.4	1.0	1.8		1.4	1.0	1.9	
**Orphanhood**											
No	615	205	33.3	Ref				Ref			
Maternal	35	10	28.6	0.8	0.4	1.7	0.3	0.8	0.4	1.8	0.5
Paternal	92	39	42.4	1.5	0.9	2.3		1.3	0.8	2.1	
Total	33	15	45.5	1.7	0.8	3.4		1.5	0.7	3.1	
Unknown	243	82	33.7	1.0	0.7	1.4		1.0	0.7	1.4	
**Food insecurity** [a]											
No	789	271	34.3	Ref				Ref			
Yes	229	80	34.9	1.0	0.8	1.4	0.9	1.2	0.8	1.7	0.5
**Sexual/pregnancy history**											
Never had sex	701	219	31.2	Ref				Ref			
Ever sex,never pregnant	158	65	41.1	1.54	1.1	2.2	0.006	1.3	0.9	1.9	0.06
Ever pregnant	159	67	42.1	1.6	1.1	2.3		1.6	1.1	2.5	

CI confidence interval; OR odds ratio; LRT likelihood ratio test

* Invited to DREAMS by 2018 defined as self-reported invitation to participate in DREAMS in 2018 (cohort enrolled in Gem in 2018) .

** Self efficacy definition: Binary outcome variable constructed based on a series of ten questions comprising a general self-efficacy scale, where a cut-off value of ≥3.5 was used to define higher self-efficacy (yes).

*** Fully adjusted model included: age group, educational attainment, socio-economic status, orphanhood, food insecurity, sexual and pregnancy history

a Food insecurity was defined based on the question: In the past 4 weeks, did you or any household member go to sleep at night hungry because there was not enough food? With responses(Yes, No)

Supplementary file 8. Conventional univariable and multivariable logistic regresssion model for the association between invited to DREAMS by 2018* with self-efficacy in 2019, showing all covariates.**

e. Nairobi

Variables at enrolment in 2017	Total N (852)	Self-efficacy n (465)	%	Unadjusted OR OR	(95% CI)		p (LRT)	Fully adjusted OR*** aOR	(95% CI)		p (LRT)
**Invited by 2018**											
No	224	113	50.4	1				1			
Yes	628	352	56.1	1.3	0.9	1.7	0.1	1.3	0.9	1.8	0.1
**Age group**											
15-17	464	247	53.2	1				1			
18-22	388	218	56.2	1.1	0.9	1.5	0.4	1.2	0.8	1.6	0.4
**Site**											
Korogocho	513	266	51.9	1				1			
Viwandani	339	199	58.7	1.3	1	1.7	0.05	1.2	0.8	1.8	0.4
**Ethnicity**											
Somali	76	29	38.2	0.4	0.3	0.7		1.0	0.3	3	
Kamba	149	79	53.0	0.8	0.5	1.2	0.04	0.8	0.5	1.2	0.9
Kikuyu	272	160	58.8	1				1			
Kisii	33	20	60.6	1.1	0.5	2.3		1.1	0.5	2.3	
Luhya	135	75	55.6	0.9	0.6	1.3		0.9	0.6	1.4	
Luo	134	78	58.2	1	0.6	1.5		1.1	0.7	1.7	
Other	53	24	45.3	0.6	0.3	1.1		1.2	0.4	3.2	
**Religion**											
Catholic	244	133	54.5	1				1			
Other Christian	466	271	58.2	1.2	0.9	1.6	<0.01	1.2	0.9	1.7	0.2
Muslim	123	48	39.0	0.5	0.3	0.8		0.5	0.2	1.5	
No religion /other	19	13	68.4	1.8	0.7	4.9		1.9	0.7	5.3	
**Food insecure** [a]											
No	564	313	55.5	1				1			
Yes	288	152	52.8	0.9	0.7	1.2	0.5	0.9	0.7	1.3	0.7
**Orphanhood**											
Not an orphan	663	360	54.3	1				1			
Single/double orphan	189	105	55.6	1.1	0.8	1.5	0.8	1	0.7	1.5	0.9
**Currently in school**											
No	312	178	57.1	1				1.0			
Yes	540	287	53.1	0.9	0.6	1.1	0.3	0.8	0.5	1.2	0.3
**Sexual/pregnancy history**											
Never had sex	557	301	54.0	1				1			
Ever sex, never preg	90	48	53.3	1	0.6	1.5	0.8	0.8	0.5	1.3	0.4
Ever pregnant	205	116	56.6	1.1	0.8	1.5		0.6	0.2	1.6	
**Ever given birth**											
No	664	357	53.8	1				1.0			
Yes	188	108	57.4	1.2	0.8	1.6	0.4	1.4	0.5	4	0.5
**Self assessed household poverty** [b]											
Very poor	115	67	58.3	1				1.0			
Moderately poor	672	358	53.3	0.8	0.6	1.2	0.3	0.8	0.5	1.2	0.2
Not poor	65	40	61.5	1.2	0.6	2.1		1.2	0.6	2.3	

**Socio-economic status**											
Poor	303	162	53.5	1				1.0			
Medium	277	141	50.9	0.9	0.7	1.3	0.1	0.9	0.6	1.3	0.5
Wealthy	272	162	59.6	1.3	0.9	1.8		1.1	0.7	1.7	
**Gender of household head**											
Male	524	285	54.4	1				1.0			
Female	328	180	54.9	1	0.8	1.4	0.9	1.0	0.7	1.4	1
**AGYW is the household head**											
No	823	447	54.3	1				1.0			
Yes	29	18	62.1	1.4	0.6	3	0.4	1.3	0.6	3.1	0.5

CI confidence interval; OR odds ratio; LRT likelihood ratio test

* Invited to DREAMS by 2018 defined as self-reported invitation to participate in DREAMS in 2017 and/or 2018.

** Self efficacy definition: Binary outcome variable constructed based on a series of ten questions comprising a general self-efficacy scale, where a cut-off value of ≥3.5 was used to define higher self-efficacy (yes).

*** Fully adjusted model included: age group, DSS study site, ethnicity, religion, food insecurity, orphanhood, in/out of school, sexual and pregnancy history, birth history, self assessed household poverty, socio-economic status, gender of the household head, whether or not the AGYW was the household head

a Food insecurity was defined as using the question: In the past 4 weeks, did you or any household member go to sleep at night hungry because there was not enough food? (yes, no)

b Self-assessed household poverty was based on the responses to the question: In your opinion, how do you assess the economic situation of your household now? (Very poor, moderately poor or not poor)

Supplementary file 8. Conventional univariable and multivariable logistic regresssion model for the association between invited to DREAMS by 2018* with self-efficacy in 2019, showing all covariates.**

f. uMkhanyakude

	Total	Self-efficacy		Unadjusted OR				Fully adjusted OR***			
**Variables at enrolment in 2017**	**N (1712)**	**n (829)**	**%**	**OR**	**(95% CI)**		**p (LRT)**	**aOR**	**(95% CI)**		**p (LRT)**
**Invited by 2018**											
No	809	384	47.5	ref				ref			
Yes	903	445	49.3	1.1	0.89	1.3	0.5	1.3	1.0	1.5	0.03
**Age group**											
13-14	414	157	37.9	ref				ref			
15-17	558	257	46.1	1.4	1.1	1.8	<0.001	1.4	1.1	1.8	<0.001
18-19	348	196	56.3	2.1	1.6	2.8		2.1	1.5	3.0	
20-22	392	219	55.9	2.1	1.6	2.7		2.2	1.5	3.4	
**Area**											
Rural	1095	545	49.8	ref				ref			
Peri-urban/urban	603	277	45.9	0.9	0.7	1.0	0.1	0.8	0.7	1.0	0.07
**Age 13-17, or 18-22 in school** [a]	1385	645	46.6	ref							
Age 13-17	972	414	42.6								
Age 18-22 and in school	391	222	56.8								
**Age 18-22, not in school and incomplete secondary education** [b]											
Yes	117	57	48.7	1.0	0.7	1.5	1	0.8	0.5	1.2	0.2
**Age 18-22, not in school and completed secondary education** [c]											
Yes	209	127	60.8	1.8	1.3	2.4	<0.001	1.2	0.8	1.7	0.4
**Socio-economic status**											
Low	592	264	44.6	ref				ref			
Middle	576	283	49.1	1.2	1.0	1.5	0.05	1.2	1.0	1.6	0.02
High	479	253	52.8	1.4	1.1	1.8		1.5	1.2	2.0	
Unknown	65	29	44.6	1.0	0.6	1.7		1.0	0.6	1.8	
**Food insecure** [d]											
No	1175	568	48.3	ref				ref			
Yes	532	260	48.9	1.0	0.8	1.3	0.8	0.9	0.7	1.1	0.4
**Migrated** [e]											
No	1432	691	48.3	ref				ref			
Yes	280	138	49.3	1.0	0.8	1.3	0.8	0.8	0.6	1.1	0.1
**Sexual & pregnancy history**											
Never had sex	1065	470	44.1	ref				ref			
Ever sex, never pregnant	218	126	57.8	1.7	1.3	2.3	<0.001	1.3	1.0	1.9	0.2
Ever pregnant	423	231	54.6	1.5	1.2	1.9		1.1	0.8	1.6	
**Experienced violence**											
No	1126	540	48.0	ref							
Yes	586	289	49.3	1.1	0.9	1.3	0.6	1.1	0.9	1.3	0.5

CI confidence interval; OR odds ratio; LRT likelihood ratio test

* Invited to DREAMS by 2018 defined as self-reported invitation to participate in DREAMS in 2017 and/or 2018.

** Self efficacy definition: Binary outcome variable constructed based on a series of ten questions comprising a general self-efficacy scale, where a cut-off value of ≥3.5 was used to define higher self-efficacy (yes).

*** Fully adjusted model included: age group, education, area, sexual & pregnancy history, socio-economic status, food insecurity, migration, violence

a Reference category for schooling dummy variables includes everyone who is in school, or 13-17 and out of school (very few, as almost all 13-17s are in school), or 18-22 and out of school with no/primary only education (few)

b Dummy variable, where 'no' (0) = all non-missing values of age and schooling, and those not coded as yes (not "18-22 not in school and incomplete secondary")

c Dummy variable, where 'no' (0) = all non-missing values of age and schooling, and those not coded as yes (not "18-22 not in school and complete secondary")

d Food insecurity was defined as any report of reducing the size of food potions or skipping meals by any member of a household because there was not enough money to buy food in the past 12 months

e Migration was defined as any movement within or outside surveillance area since age of 13
