# Supplementary material for: Impact of the DREAMS Partnership on social support and general self-efficacy among adolescent girls and young women: causal analysis of population-based cohorts in Kenya and South Africa
Source: BMJ Glob Health. 2022 Mar 1;7(3):e006965. doi: 10.1136/bmjgh-2021-006965 (PMC8889325; doi:10.1136/bmjgh-2021-006965)
Supplement: Supplementary data [file bmjgh-2021-006965supp009.pdf]

Supplementary file 9(a). Sensitivity analyses alternative methods to estimate causal effect of DREAMS on social support in 2019, among all AGYW followed up in 2019

	% Social support in total study population	Estimated % social support if none benefit from DREAMS	95% CI	Estimated % social support if all benefit from DREAMS	95% CI	Difference in estimated %	95% CI
**Gem**							
**Age 13-22 years at cohort enrolment**							
PS-regression adjustment*	40.4	35.5	31.1,39.6	43.3	39.3,47.3	7.8	2.1,14.0
PS-stratification	40.4	35.9	31.2,40.5	43.6	39.5,47.4	7.6	1.8,13.9
PS-inverse probability of treatment weighting	40.4	35.8	31.4,40.0	43.3	39.7,47.3	7.5	0.9,13.4
Counterfactual framework multivariable outcome regression	40.4	35.6	31.0,40.2	43.4	39.3,47.1	7.8	1.7,13.6
**Age 13-17 years at cohort enrolment**							
PS-regression adjustment*	37.0	31.2	26.0,37.3	40.7	35.7,46.3	9.5	1.9,17.3
Counterfactual framework multivariable outcome regression	37.0	30.5	25.1,36.4	41.2	36.1,46.1	10.6	3.3,18.0
**Age 18-22 years at cohort enrolment**							
PS-regression adjustment*	44.9	43.5	35.7,51.1	48.2	41.6,55.0	4.7	-4.5,14.1
Counterfactual framework multivariable outcome regression	44.9	43.6	36.0,51.0	46.9	39.9,53.1	3.3	-6.7,13.5
**Nairobi**							
**Age 15-22 years at cohort enrolment**							
PS-regression adjustment*	56.3	49.4	42.6,56.2	58.2	54.1,62.3	8.8	1.2,16.7
PS-stratification	56.3	48.4	41.4,55.4	58.3	54.2,62.2	9.8	1.4,17.4
PS-inverse probability of treatment weighting	56.3	49.1	42.3,56.2	58.2	54.1,62.2	9.1	1.3,17.1
Counterfactual framework multivariable outcome regression	56.3	49.8	43.1,56.9	58.2	54.1,62.3	8.4	0.4,16.4
**Age 15-17 years at cohort enrolment**							
PS-regression adjustment*	57.3	46.1	36.2,55.6	60.0	54.6,65.0	14.0	3.0,25.0
Counterfactual framework multivariable outcome regression	57.3	47.2	37.6,57.0	59.6	54.1,64.5	12.3	1.0,23.5
**Age 18-22 years at cohort enrolment**							
PS-regression adjustment	55.2	53.3	43.9,61.3	56.0	49.5,62.5	2.6	-7.3,14.1
Counterfactual framework multivariable outcome regression	55.2	52.8	43.5,61.3	56.6	50.3,62.9	3.8	-6.7,15.1

**uMkhanyakude**							
**Age 13-22 years at cohort enrolment**							
PS-regression adjustment*	45.4	44.2	40.5,47.7	46.0	42.5,49.5	1.8	-3.2,6.6
PS-stratification	45.4	44.1	40.4,47.4	46.0	42.6,49.7	1.9	-2.8,6.9
PS-inverse probability of treatment weighting	45.4	44.0	40.4,47.4	45.7	42.1,49.2	1.7	-3.2,6.4
Counterfactual framework multivariable outcome regression	45.4	44.0	40.4,47.6	45.9	42.3,49.3	1.8	-3.0,6.8
**Age 13-17 years at cohort enrolment**							
PS-regression adjustment*	45.4	43.2	38.0,48.1	46.7	42.7,50.7	3.6	-2.6,10.2
Counterfactual framework multivariable outcome regression	45.4	42.6	37.4,47.8	46.7	42.7,50.7	4.1	-2.1,10.5
**Age 18-22 years at cohort enrolment**							
PS-regression adjustment*	45.5	45.6	41.2,50.4	44.9	39.1,50.4	-0.6	-7.5,6.6
Counterfactual framework multivariable outcome regression	45.5	45.9	41.6,50.8	44.7	38.9,50.2	-1.2	-8.1,5.9

PS : propensity score

Outcome definitions: Binary outcome variable constructed where a high level of social support was defined as a ‘yes’ response to at least three out of four questions: “Is there a female in your community from whom you can borrow money in an emergency?; “Do you have at least one trusted female friend?”; “Do you know a woman in your community, other than a mother or guardian, whom you could turn to if you had a serious problem?”; “Do you have a safe and private place to meet with girls and young women who are like you?”

**Primary approach & main result reported in table 3*

Supplementary file 9(b): Sensitivity analyses (alternative methods) to estimate causal effect of DREAMS on self efficacy in 2019, among all AGYW followed up in 2019

	% Self efficacy in total study population	Estimated % self efficacy if none benefit from DREAMS	95% CI	Estimated % self efficacy if all benefit from DREAMS	95% CI	Difference in estimated %	95% CI
**Gem**							
**Age 13-22 years at cohort enrolment**							
PS-regression adjustment*	34.5	31.8	27.3,36.0	35.6	31.1,39.0	3.8	-4.0,9.1
PS-stratification	34.5	32.5	27.7,36.2	35.3	31.3,39.6	2.8	-2.3,8.6
PS-inverse probability of treatment weighting	34.5	32.3	27.2,36.8	35.8	32.7,40.4	3.5	-1.8,11.2
Counterfactual framework multivariable outcome regression	34.5	31.9	27.2,36.6	35.6	31.2,39.8	3.7	-2.2,10.4
**Age 13-17 years at cohort enrolment**							
PS-regression adjustment*	30.1	27.0	21.6,31.7	32.8	28.2,37.8	5.7	-0.1,13.4
Counterfactual framework multivariable outcome regression	30.1	25.6	20.8,31.9	32.7	28.5,37.2	7.1	0.8,13.3
**Age 18-22 years at cohort enrolment**							
PS-regression adjustment*	41.4	41.0	33.6,49.8	41.2	34.2,46.6	0.2	-11.7,9.6
Counterfactual framework multivariable outcome regression	41.4	41.8	35.5,49.8	40.1	31.6,46.3	-1.7	-13.0,8.8
**Nairobi**							
**Age 15-22 years at cohort enrolment**							
PS-regression adjustment*	54.6	50.5	43.8,58.2	56.3	52.2,60.2	5.7	-2.7,13.9
PS-stratification	54.6	49.2	42.7,57.3	56.4	52.3,60.3	7.1	-2.2,14.6
PS-inverse probability of treatment weighting	54.6	49.8	42.9,57.3	56.4	52.3,60.3	6.6	1.5,14.8
Counterfactual framework multivariable outcome regression	54.6	51.1	44.4,58.7	56.1	52.2,60.0	5.0	-3.2,12.9
**Age 15-17 years at cohort enrolment**							
PS-regression adjustment*	53.2	50.6	41.3,61.2	53.9	49.0,59.0	3.3	-8.7,14.3
Counterfactual framework multivariable outcome regression	53.2	52.9	43.1,63.2	53.8	48.6,58.8	0.9	-10.8,12.2
**Age 18-22 years at cohort enrolment**							
PS-regression adjustment	56.2	50.5	41.9,59.5	59.1	53.2,65.5	8.6	-3.0,19.8
Counterfactual framework multivariable outcome regression	56.2	49.0	40.5,58.9	59.0	53.1,65.6	10.0	-0.9,20.6

**uMkhanyakude**							
**Age 13-22 years at cohort enrolment**							
PS-regression adjustment*	48.4	45.5	42.1,49.3	51.0	47.4,54.4	5.4	0.5,10.1
PS-stratification	48.4	45.9	42.5,49.7	50.9	47.5,54.2	4.9	0.2,9.7
PS-inverse probability of treatment weighting	48.4	45.6	42.1,49.3	50.8	47.2,54.1	5.2	0.3,9.7
Counterfactual framework multivariable outcome regression	48.4	45.5	42.0,49.3	50.8	47.3,54.2	5.3	0.3,9.8
**Age 13-17 years at cohort enrolment**							
PS-regression adjustment*	42.6	38.7	33.6,44.1	45.3	41.4,49.7	6.7	0.3,12.6
Counterfactual framework multivariable outcome regression	42.6	38.6	33.6,44.0	45.5	41.5,49.8	6.9	0.5,12.9
**Age 18-22 years at cohort enrolment**							
PS-regression adjustment*	56.1	54.5	49.9,59.4	58.3	52.2,64.0	3.8	-3.6,11.2
Counterfactual framework multivariable outcome regression	56.1	54.6	50.1,59.5	57.8	51.4,63.3	3.2	-4.5,10.7

PS : propensity score

Outcome definitions: Binary outcome variable constructed based on a series of ten questions comprising a general self-efficacy scale, where a cut-off value of ≥3.5 was used to define high self-efficacy.

**Primary approach & main result reported in table 3*
